# Population genomics of finless porpoises reveal an incipient cetacean species adapted to freshwater

**DOI:** 10.1038/s41467-018-03722-x

**Published:** 2018-04-10

**Authors:** Xuming Zhou, Xuanmin Guang, Di Sun, Shixia Xu, Mingzhou Li, Inge Seim, Wencai Jie, Linfeng Yang, Qianhua Zhu, Jiabao Xu, Qiang Gao, Alaattin Kaya, Qianhui Dou, Bingyao Chen, Wenhua Ren, Shuaicheng Li, Kaiya Zhou, Vadim N. Gladyshev, Rasmus Nielsen, Xiaodong Fang, Guang Yang

**Affiliations:** 1 Jiangsu Key Laboratory for Biodiversity and Biotechnology, College of Life Sciences, Nanjing Normal University, 210023 Nanjing, China; 2 Division of Genetics, Department of Medicine, Brigham and Women’s Hospital, Harvard Medical School, Harvard Medical School, Boston, MA 02115 USA; 3 BGI-Shenzhen, 518083 Shenzhen, China; 4 The Key Laboratory of Conservation Biology for Endangered Wildlife of the Ministry of Education, College of Life Sciences, Zhejiang University, 310058 Hangzhou, China; 5 Institute of Animal Genetics and Breeding, College of Animal Science and Technology, Sichuan Agricultural University, 611130 Chengdu, China; 6 Comparative and Endocrine Biology Laboratory, Translational Research Institute-Institute of Health and Biomedical Innovation, School of Biomedical Sciences, Queensland University of Technology, Woolloongabba, QLD 4102 Australia; 7 Department of Computer Sciences, City University of Hong Kong, 999077 Hong Kong, China; 8 Department of Integrative Biology, University of California, Berkeley, CA 94720 USA

## Abstract

Cetaceans (whales, dolphins, and porpoises) are a group of mammals adapted to various aquatic habitats, from oceans to freshwater rivers. We report the sequencing, de novo assembly and analysis of a finless porpoise genome, and the re-sequencing of an additional 48 finless porpoise individuals. We use these data to reconstruct the demographic history of finless porpoises from their origin to the occupation into the Yangtze River. Analyses of selection between marine and freshwater porpoises identify genes associated with renal water homeostasis and urea cycle, such as urea transporter 2 and angiotensin I-converting enzyme 2, which are likely adaptations associated with the difference in osmotic stress between ocean and rivers. Our results strongly suggest that the critically endangered Yangtze finless porpoises are reproductively isolated from other porpoise populations and harbor unique genetic adaptations, supporting that they should be considered a unique incipient species.

## Abstract (web summary)

Whales, dolphins and porpoises are adapted to various aquatic habitats. Here, Zhou et al. show that polymorphisms associated with renal function and the urea cycle have undergone selection in the freshwater Yangtze finless porpoise and provide genomic evidence of incipient speciation.

## Introduction

Whales, dolphins, and porpoises are collectively termed cetaceans. Having evolved from terrestrial ancestors to occupy aquatic niches^1^, these mammals fascinate scientists and the public alike. The finless porpoise (*Neophocaena* spp.) is a group of small cetaceans, which were characterized by spade-shaped teeth, a short, blunt snout and lack of a true dorsal fin, inhabiting coasts of southern and eastern Asia. Some molecular evidence placed finless porpoises as the most basal clade of extant porpoises (family Phocoenidae)^2–5^. After originating from a marine ancestor, finless porpoises gradually evolved to inhabit different aquatic niches. For example, in China, its distribution covers all coastal waters and particularly, one population dispersed into the Yangtze River and established the sole freshwater porpoise population in the world (i.e., the Yangtze finless porpoise^6–8^), which makes it an ideal natural model to study the evolutionary adaptation of cetaceans to freshwater. Moreover, the Yangtze finless porpoise is likely the sole cetacean living in the Yangtze River as another river dolphin, the “white-flag” dolphin or baiji (*Lipotes vexillifer*), has been recognized as functionally extinct since large-scale surveys conducted in 2006 sighted no individual in the wild^9^. Recent surveys indicated that the population size of Yangtze finless porpoise is around 1000 individuals^10^, and it is experiencing an annual population decline of ~13.7%^11^. It has, therefore, since 2013 been considered critically endangered by the IUCN^12^ and may eventually go extinct, if effective measures of conservation are not urgently implemented.

Finless porpoises have traditionally been classified into a single species, *N*. *phocaenoides*, with three geographic populations or subspecies, that have colonized freshwater (Yangtze River) and saltwater environments along the coasts of the Indo-Pacific Ocean^13^. Previous analyses of different markers (e.g., mtDNA haplotypes and morphological variables such as the structure of the dorsal ridge, tubercled area, etc.) proposed two models for the taxonomy of finless porpoises: (i) the three subspecies hypothesis, which assigned the finless porpoises to a single species with three subspecies (wide-ridged *N*. *p*. *phocaenoides*, narrow-ridged *N. p*. *asiaeorientalis*, and *N*. *p*. *sunameri*)^6, 7^, and (ii) the two species hypothesis that invoked two distinct species, Indo-Pacific finless porpoises *N. phocaenoides* (the wide-ridged form) and narrow-ridged finless porpoises *N. asiaeorientalis*^8^, the latter of which includes two subspecies (*N*. *a*. *asiaeorientalis* endemic to the mainstream Yangtze River and the river-communicating lakes of China, and *N. a*. *sunameri* from the Yellow/Bohai Sea, northern East China Sea, and Japan Sea). A primary objective of this paper is to resolve this issue using whole-genome sequencing in order to facilitate current and future conservation efforts.

Here, we apply a whole-genome shotgun strategy to sequence the genome of a finless porpoise to a depth of 106× (~265.5 Gb) (Supplementary Table 1). We also sequenced the genomes of an additional 48 finless porpoises from different geographical regions, at a depth of ~10× to 30× for each individual (~1.87 trillion base in total). Our analyses strongly suggest that Yangtze finless porpoises are genetically isolated from other porpoise populations and reveal the genomic signatures of adaptation to the freshwater environment of this incipient species.

## Results

### Finless porpoise genome assembly and genomic variation

The finless porpoise genome assembly was approximately 2.30 Gb in length (scaffold), with contig and scaffold N50 values of 26.7 Kb and 6.3 Mb, respectively (Supplementary Table 2). Characterization of GC content, repeats diversity and noncoding RNAs prediction can be found at Supplementary Note 1, Supplementary Tables 3–13, and Supplementary Figs. 1–3. We employed homology and de novo methods, combined with blood transcriptome sequencing (RNA-seq) data, to predict 22,014 protein-coding genes in the finless porpoise genome (93.0% were functionally classified, Supplementary Tables 8 and 9). Using these protein-coding genes, we generated a timescale for mammalian evolution and identified 57 positively selected genes (PSGs) in the finless porpoise using a branch site model^14^ (Supplementary Fig. 4, Supplementary Table 14, and Supplementary Note 2).

**Fig. 1:**
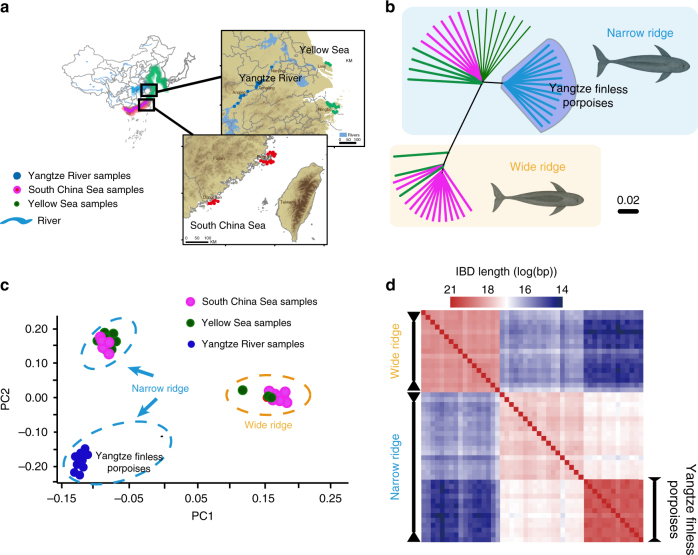
Phylogeny and population structure of finless porpoises. **a** Current geographic distribution of the finless porpoise. The map was retrieved from http://www.naturalearthdata.com (Public Domain; date accessed: Feb 2017) and generated using ArcGIS 9.3^62^. **b** Neighbor-joining tree constructed from the allele-sharing matrix of variants of 48 finless porpoises. The finless porpoise images were created by Chen Yu in Adobe Photoshop CS6. **c** Principal components analysis (PCA) of 48 finless porpoises. In particular, the first eigenvector separate the two main morphological forms, i.e., the wide-ridged form and narrow-ridged (variance explained = 12.92%, Tracy–Widom *P* = 3.26 × 10^−11^), and the second eigenvector separate the Yangtze River population from the other narrow-ridged individuals (variance explained = 2.53%, Tracy–Widom *P* = 8.72 × 10^−50^). **d** Estimated haplotype sharing in the finless porpoises. The heat map colors represent the total length of IBD blocks for each pairwise comparison

To gain a better understanding of the phylogenetic relationships and evolutionary history of the members of this species-complex, we sequenced 48 finless porpoises to an average sequencing depth of ~14.5× per individual resulting in 98.6% sequencing coverage. The 48 wild finless porpoises samples collected and sequenced here represent the main natural populations of finless porpoises from the middle and lower reaches of the Yangtze River and the coastal China seas (Fig. 1a, Supplementary Fig. 65, and Supplementary Tables 15 and 16). After applying stringent quality control criteria (Methods, Supplementary Note 3), we identified a total of 13.3 million (M) SNPs in the finless porpoise populations (Supplementary Tables 16–18 and Supplementary Fig. 6).

### Phylogeny and population structure of finless porpoises

We first clustered individuals using average genomic distance by both phylogenetic reconstruction and principal components analysis (PCA)^15^. Both analyses provided strong support for the subdivision of finless porpoises in Chinese waters into three distinct groups (Fig. 1b, c, Supplementary Figs. 7, 8, and Supplementary Table 19). Particularly, the first cluster contained the individuals with wide ridge from the South China Sea and Yellow Sea, the second cluster was mainly comprised of all narrow-ridge individuals from Yellow/Bohai Sea, and South China Sea (Ningbo, Pingtan and Lvshi), and all individuals from the Yangtze River were grouped into a distinct group within narrow-ridged cluster. Varying the number of ancestry components (*K*) using frappe^16^ also recapitulated these findings (Supplementary Fig. 9). As an example, with *K* = 2, we observed a division between wide-ridged form and narrow-ridged form finless porpoises. When *K* = 3, individuals from the Yangtze River were further separated from the narrow-ridged form cluster; this finding was corroborated by cross-validation and was generally consistent with the phylogeny and biogeographic distribution (Supplementary Fig. 10). Measures of haplotype identity-by-descent (IBD) (Fig. 1d and Supplementary Fig. 11) and linkage disequilibrium (LD) (Supplementary Fig. 12) further supported that larger haplotypes segments were shared among Yangtze River porpoises.

### Demographic history and species delimitation

**Fig. 2:**
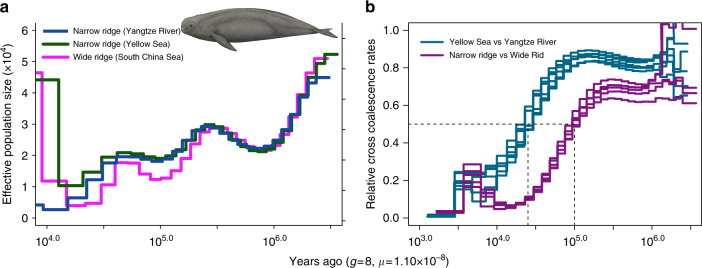
Demographic histories of finless porpoises. **a** Demographic histories of finless porpoises using the PSMC model. *g* (generation time) = 8 years; *μ* (neutral mutation rate per generation) = 1.10 × 10^−8^. The finless porpoise image was created by Chen Yu in Adobe Photoshop CS6. **b** Relative cross coalescence rates (CCR) between finless porpoise populations. Wide-ridged/narrow-ridged form pairs are shown in pink colors, pairs within narrow-ridged forms (Yangtze River/Yellow Sea populations pairs) in blue colors. The relative cross coalescence rate is close to one when the two populations are well mixed, and zero after they have fully split

To reconstruct the demographic history of finless porpoises, we first employed the pairwise sequentially Markovian coalescent (PSMC) method^17^. Unsurprisingly, we found that the effective population sizes (*N*_e_) of the three major groups are highly correlated until ~20 k years ago. We observed two peaks of *N*_e_ at ~0.5 million years ago and ~60 thousand years ago and a reduction in population size ~0.1 million years ago (Fig. 2a, Supplementary Fig. 13, and Supplementary Note 4). Narrow-ridged finless porpoises showed relative higher *N*_e_ than wide-ridged forms from 0.5 million years ago to 20 thousand years ago and the *N*_e_ difference of two forms achieved its maximum at ~100 thousand years ago (1.95 × 10^4^ ± 0.06 × 10^4^ individuals in “marine” wide-ridged vs. 1.27 × 10^4^ ± 0.04 × 10^4^ individuals in narrow-ridged finless porpoises). The split of *N*_e_ curves within narrow-ridged finless porpoises occurred ~20 thousand years ago, perhaps suggesting a divergence of the populations at this time, or perhaps resulting from the well-known difficulties in estimating recent values of *N*_e_ using the PSMC method. We further inferred split times among populations based on relative cross-coalescent rates (RCCR) using a multiple sequentially Markovian coalescent approach (MSMC)^18^. The MSMC curves are compatible among different individuals when comparing narrow-ridged to wide-ridge formed porpoises, and also when comparing Yangtze River to Yellow Sea narrow-ridged porpoises (Fig. 2b). The RCCR curves converged to zero for recent times, and showed no evidence of cross-coalescences within the last 5 kya, indicating no current gene flow between the population groups. The curves reached a value of 0.5 at ~40 kya and 100 kya ago for the two comparisons, respectively (Fig. 2b), and converged to their maximum values at about 100 kya and 500 kya, respectively. A previous study suggested that the ancestral population of finless porpoise was interrupted by a land bridge connecting Taiwan and mainland China during the LGM ~18 thousand years ago^19^. Our analysis, which provided a much older estimation of the divergence of finless porpoises, suggested instead that Pleistocene glaciation may have contributed to the isolation of ancient finless porpoises. The divergence of the Yangtze River population from other narrow-ridged individuals between 5000 and 40,000 years ago may suggest that sea-level oscillation before and after LGM could have contributed to the isolation of the freshwater population. Also, analyses using f3-statistics^20^ indicated no ancient admixture in the Yangtze River population and other finless porpoises (f3-statistics *=* 0.059, *Z*-scores = 123.7) (Supplementary Table 20). The complete reproductive isolation of the three groups of finless porpoises was also supported by a species delineation analysis using Bayes factors^21^, which provided strong statistical support for a “three-species model” (marginal log likelihood = −5.7 × 10^6^) rather than a “two-species model” (marginal log likelihood = −6.9 × 10^6^) (Supplementary Table 21).

### Signatures of positive selection in hypoxia-associated genes

**Fig. 3:**
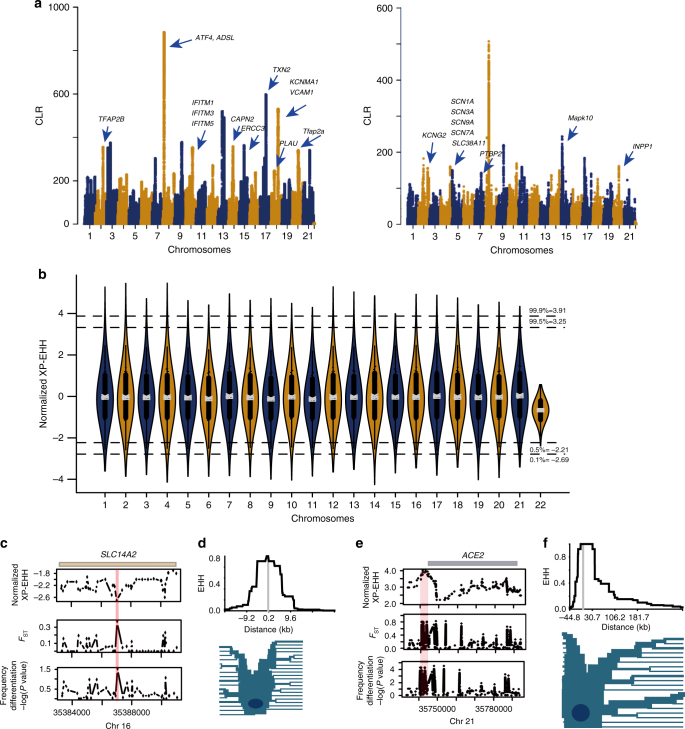
Identification of selective sweeps in finless porpoise genomes. **a** Selective sweep regions identified in the narrow-ridged and wide-ridged finless porpoises. Plot of composite likelihood ratio (CLR) values that estimated in the wide-ridge (left panel) and narrow-ridge (right panel) finless porpoises. Genes residing within the selected regions that functioned in neuronal processes and response to biotic stimulus are presented for each peak according to their locations. **b** Violin plots of normalized XP-EHH values were generated by comparing the Yangtze River population and their marine relatives (Yellow Sea population) (the medians are shown). **c** Normalized XP-EHH values, population differentiations (*F*_ST_), and frequency differences of the selected region near gene *SLC14A2*. **d** The EHH at varying distances from the core region (upper panel) and Haplotype bifurcation diagrams (lower panel) for core haplotype at *SLC14A2*. **e** Normalized XP-EHH values, population differences (*F*_ST_), and frequency differences of the selected region near gene *ACE2*. **f** The EHH at varying distances from the core region (upper panel) and Haplotype bifurcation diagrams (lower panel) for core haplotype at *ACE2*

Despite being sympatric in the Taiwan Strait and surrounding waters, the two major forms of finless porpoise are highly morphologically differentiated. For example, in the wide-ridged finless porpoises, the greatest width of the tuberculed area varies from 4.0 to 10.0 cm and there are between 10 and 18 rows of tubercules, whereas in narrow-ridged finless porpoises the width of the tuberculed area is between 0.3 and 0.7 cm and there are only 3–5 rows of tubercules^8, 19^. To detect genomic footprints of positive selection in finless porpoises that might be related to these differences, we used the recent improved version of the composite likelihood ratio (CLR) test^22^ implemented in SweepFinder2^23^. We identified 218 genes and 144 candidate genes embedded in 39 CLR peaks with strong selective sweep signals in wide- and narrow-ridged form populations, respectively (Fig. 3a, Supplementary Tables 22, 23, and Supplementary Note 5). Those candidate genes are significantly enriched in biological process such as regulation of postsynaptic membrane potential (*P* = 1.30 × 10^−5^, Fisher's exact test), neuronal action potential (*P* = 3.60 × 10^−5^, Fisher's exact test), neuropeptide signaling pathway (*P* = 2.10 × 10^−2^, Fisher's exact test), and response to biotic stimulus (*P* = 3.70 × 10^−4^, Fisher's exact test) (Supplementary Tables 24, 25). This is to some degree consistent with the proposed sensory function of tubercles, as they possess an abundance of nerve endings^24^.

The genomic region with the highest CLR in wide-ridged porpoises was embedded with six intergenic, three upstream, one downstream, and one intronic mutations of *Atf4*, which is a transcription factor induced by environmental stress including nutrient stress^25^ and hypoxia^26^. Further examination of the candidate genes reveals seven genes (*KCNMA1*, *VCAM1*, *TXN2*, *ADSL*, *CAPN2*, *ERCC3*, and* PLAU*) associated with response to hypoxia (*P* = 2.10 × 10^−3^, Fisher's exact test) in the wide-ridged form of finless porpoises. The possible connection to hypoxia is interesting, because the typical habitats of two forms finless porpoises differ drastically in terms of sea level. In particular, narrow-ridged finless porpoises is found in temperate coastal waters from the Taiwan Strait northwards to Korea and central Japan, including the middle and lower reaches of the Yangtze River, which are generally shallow ocean regions and almost entirely less than 150 m deep. However, the average sea level for habitats of wide-ridged form finless porpoises (the tropical and subtropical waters of the Indian Ocean and Southeast Asia) is comparatively deeper. For example, the South China Sea is 1212 m deep, with more than 5500 m at its deepest. Nevertheless, it is noteworthy that both narrow- and wide-ridged forms of finless porpoises are sympatric in the Taiwan Strait. Future experimental studies aimed at exploring physiological differences between these species are vital for understanding possible adaptions to hypoxia in finless porpoises.

### Genetic adaptation to freshwater of Yangtze River porpoises

Finless porpoises have colonized both freshwater and saltwater, and our data suggested that the freshwater Yangtze River population emerged 5000–100,000 years ago. To identify candidate mutations that may have been under positive selection specifically in the Yangtze River population or the marine populations of the narrow-ridged finless porpoise, we used the XP-EHH tests of neutrality^27^. Employing a normalized XP-EHH threshold of 3.91 (top 0.1%) and −2.70 (lowest 0.1%), we identified 6035 candidate SNPs in the Yangtze finless porpoises (83 genes) and their marine counterparties (187 genes) (Fig. 3b, Supplementary Data 1, Supplementary Data 2, and Supplementary Note 5). Interestingly, these genes were significantly enriched in “kidney development” (*P* = 4.10 × 10^−4^, Fisher's exact test) and “ureteric bud development” (*P* = 5.70 × 10^−3^, Fisher's exact test) (Supplementary Tables 26 and 27), which could contribute to the distinct kidney structures of freshwater and marine finless porpoises^28^. For example, three genes (*ADCY1*, *DYNC2H1*, and *SLC14A2*) associated with the gene ontology terms “renal water homeostasis,” “vasopressin-regulated water reabsorption,” and “urea transport” were candidates in the marine porpoises. One of them, *SLC14A2*, which harbored one intronic candidate SNP, is a key protein that encodes one of the two urea transporters in mammals that interacts with vasopressin and regulates the plasma urea^29^. Positive selection of this gene in cetaceans has previously been described^30^. In addition, analyses of the renal transcriptome have revealed differential expression of *SLC14A2* between freshwater and marine finless porpoises^31^. The selection signature (Fig. 3c, d) on these genes is consistent with previous studies that have shown a significantly higher urine but lower serum urea in East Asian porpoises (urine: 74.96 ± 20.06 mM L^−1^, serum: 14.35 ± 3.11 mM L^−1^) than in Yangtze finless porpoises (urine: 40.7 ± 15.78 mM L^−1^, serum: 16.75 ± 3.19 mM L^−1^) and other marine cetaceans^32^. Similarly, the urine osmolality of Yangtze finless porpoises (866 mosM) was previously found to be statistically lower than that of their marine counterparts (1645 mosM)^28^, consistent with their living in the hypotonic freshwater environment.

In the freshwater finless porpoises, a top candidate gene is *ACE2* (associated with 6 intergenic candidate SNPs of which one has a normalized XP-EHH = 4.13, *F*_ST_ = 0.70, and frequency *P* value = 0.0003) (Fig. 3e, f). This gene also ranked among the top 0.12% of genes (represented by a SNP with normalized XP-EHH = 3.85, *F*_ST_ = 0.56, and frequency *P* value = 0.002) in a comparison between freshwater porpoises and all Yellow Sea porpoises. The protein encoded by *ACE2* is a monocarboxyl peptidase that functions in the renin–angiotensin system and plays important roles in cardiovascular, renal, pulmonary, and central nervous systems^33^. Loss of the *ACE2* gene in mice leads to progressive kidney injury and increased urinary albumin excretion^34^. Additionally, the development of glomerular injury and an increase in urinary albumin/creatinine ratio was found in mice treated with the inhibitor of *ACE2*^35^. Similarly to *SLC14A2*, *ACE2* show multiple cetacean-specific changes^36^, suggesting that evolution of this gene is crucial for adaption of cetaceans to different habitats. Adaptation to freshwater driven by genetic variants regulating kidney function has previously been observed in other marine and freshwater sticklebacks^37^.

## Discussion

Our analyses have shown that the three main groups of finless porpoises are reproductively isolated and have not shared gene flow for thousands of years. They show unique individualized signatures of genetic adaptation to different environments. In comparisons of narrow-ridge and wide-ridged finless porpoises, we found a number of candidate genes related to hypoxia showing strong evidence of selection, possibly a consequence of the difference in sea-level in the environments inhabited by these two groups. The genetic differentiation between two groups is perhaps not surprising given that they also are easily distinguishable by a number of morphological features. It might be more surprising that the Yangtze River populations and the marine populations of the narrow-ridge finless porpoise show many signatures of selection relating to renal function, highly likely related to differential adaptation to marine and freshwater habitats. Although the Yangtze finless porpoise was first treated as a separate species in 1970s^38, 39^, it has not been accepted by most other researchers so far, and nearly all subsequent studies supported the subspecies status of the Yangtze finless porpoise in *Neophocaena phocaenoides* or *Neophocaena asiaeorientalis*^2, 40, 41^. However, the present findings of significant population differentiation, lack of gene flow, and unique adaptive divergence in the Yangtze finless porpoise make it clear that the Yangtze finless porpoise is genetically and reproductively isolated from its marine counterpart and thus represent an incipient species. Thus, the classification of the narrow-ridged finless porpoises into two species, i.e., the Yangtze finless porpoise *Neophocoena asiaeorientalis* Pilleri and Gihr, 1972 and the East Asia finless porpoise *Neophocoena sunameri* Pilleri and Gihr, 1972, should be restored. The Yangtze finless porpoise, *Neophocoena asiaeorientalis*, is a distinct endemic Chinese species inhabiting the Yangtze River, and is a new flagship species for conserving the biodiversity and the aquatic ecosystem of the Yangtze River.

## Methods

### Samples and data

All finless porpoises that were used for de novo sequencing and whole-genome sequencing have been preserved at Nanjing Normal University at −20 °C. They were all killed incidentally in fishing nets or were found stranded between 1979 and 2009, before the initiation of this research project. Genomic DNA was extracted from skeletal muscles. The de novo assembly of a male finless porpoise (narrow ridged) was sequenced at 106× depth coverage and the whole genomes of additional 48 finless porpoises were sequenced at intermediate depth coverage (an average of ~13×) (see Supplementary Note 1). All DNA libraries were sequenced on the Illumina HiSeq 2000 platform. After filtering out the adapter sequences, low-quality reads and duplicate reads, a total of 265.5 Gb of high-quality data, were used for genome assembly. The fresh blood from two finless porpoise individuals (narrow ridged) for RNA-seq sequencing were sampled from the Tongling Freshwater Porpoise National Nature Reserve (Tongling City, China) and was approved by the Nanjing Normal University Animal Care Committee. Two sequencing libraries were constructed using an Illumina standard mRNA-Seq Prep Kit and the sequencing was performed on Illumina HiSeq 2000 platform following the manufacturer’s instructions. A detailed description of genome size estimation, assembly, annotation, gene structure prediction (based on homology, de novo prediction and RNA-seq data) and functional annotation, and RNA-seq is included in the Supplementary Note 1.

### Gene family and positively selected genes

All mammalian genomes with significant sequence coverage from the current Entrez Genome Project at NCBI were used in this study (a total of 24 organisms). The genome sequence of the bowhead whale was retrieved from The Bowhead Whale Genome Resource (http://www.bowhead-whale.org/). Gene families were constructed using TreeFam^42^. The phylogenetic tree was reconstructed using the 3911 single-copy genes shared by the finless porpoises, 6 other cetaceans and 10 other mammals using the maximum-likelihood algorithm as implemented in RAxML software^43^. The divergence times for the taxa were estimated by Reltime^44^ on the basis of fourfold degenerate codon sites. The branch site model^14^ was used to detect positive selection along a target branch. *P* value of each gene was computed using the likelihood ratio tests (LRTs) and all gene alignment with *P* value less than 0.05 were manually checked.

### Reads mapping and variant calling

The high-quality reads were aligned to finless porpoise assembly using BWA mem software^45^ and the alignment was processed using a Bayesian approach as implemented in the package SAMtools (version 1.2)^46^. Variant calling was performed following the standard pipeline through Picard (http://broadinstitute.github.io/picard) and the Genome Analysis Toolkit (GATK, v3.4)^47^ with the analysis type of HaplotypeCaller based method of HMM likelihood function. To determine pseudo-chromosome position for SNPs, the scaffolds of finless porpoise were mapped and assembled onto the chromosomes based upon their syntenies with the cattle genome (UMD3.1).

### Phylogenetics and population structure

Phylogenetic trees of 48 finless porpoise individuals were reconstructed using neighbor-joining (NJ) method implemented in VCF-kit (http://vcf-kit.readthedocs.io/) and maximum-likelihood (ML) method using RAxML (version 7.2.3)^48^. The principal component analysis (PCA) was conducted using EIGENSOFT3.0 and the significance of eigenvectors were determined with the Tracey–Widom test^15^. FDIST^49^ was used to determine presumably neutral SNPs for PCA analyses and phylogeny reconstruction. Population structure was inferred by ADMIXTURE^50^ with tenfold cross-validation. The ancestral sequences of finless porpoises were inferred using the program frappe^16^ without assuming any prior information about their structure. Identical-by-descent (IBD) haplotypes was estimated using BEAGLE^51^ and linkage disequilibrium (LD) pattern and the coefficient of determination between any two loci was calculated using Haploview for each population or genetic cluster^52^.

### Demographic history reconstruction and gene flow

The PSMC model was utilized to estimate changes in effective population size using heterozygous sites across the genome and this method has been widely used in other mammals^17, 53–56^. The estimated TMRCA (time to the most recent common ancestor) is in units of 2*N*_0_ time and the mean generation time was set at 8 years and *μ* (unit: nt/generation) was estimated as 1.10 × 10^−8^. SNPs located in scaffolds homologous to cow X chromosomal regions were excluded and the remaining SNPs were used for PSMC analyses and estimation of the relative cross coalescence rates^18^. The f3-statistics was implemented in ADMIXtools^20^. Bayes factor delimitation was employed in SNAPP^57^ and BEAST^58^ by estimating the marginal likelihood^59^ for each competing species delimitation model, and then Bayes factors^21, 60^ was used to assess support for comparing models. The path sampling analyses for each model included 20 steps and tree sampled 100,000 generations with a burn-in of 8000 generations.

### Detecting SNPs under selection

To investigate the selection signals of wide-ridged and narrow-ridged forms of finless porpoises, we first scanned the genome for target regions of positive selection using Sweepfinder2^22, 23^, which uses local deviations of the site frequency spectrum (SFS). The composite likelihood ratio (CLR) was estimated for each SNP and the top 20 genome “peaks” with a CLR higher than 0.2% of all CLRs were used to define sweep candidates. Genes that overlapped with or were near (within 1 Mb flanking the sweep region) were extracted. We also identified selected regions across the genome between Yangtze and marine finless porpoises using the XP-EHH tests of neutrality as implemented in the Selscan package^61^ and Sweep^27^. SNPs were pruned for minor allele frequency (MAF) >1% in each population. The XP-EHH values were normalized by subtracting the genome-wide mean XP-EHH and dividing by the standard deviation. Top XP-EHH value was selected and if it falls into top 0.1% of XP-EHH values, the window was selected for genes in the 5-kb flanking region around the highest XP-EHH value. In order to estimate the difference of allele frequency for each allele (*i*) in two compared groups (for example, groups A and B), we first calculated the accumulated distance of allele frequency in A and B groups ${T}_{\it{d}} = \mathop {\sum}\nolimits_{{i = 1}}^{n} {({\mathrm{Freq}}\,{\mathrm{A}}{i} - {\mathrm{Freq}}\,{\mathrm{B}}{i})} ^2$. Then, the relative contribution of allele was determined and a *P* value was calculated for each SNP based on the empirical distribution of its relative contribution.

### Data availability

The data discussed in this publication have been deposited in NCBI’s short read archive under the accession number SRP090345. This Whole Genome Shotgun project has been deposited at DDBJ/ENA/GenBank under the accession number MKKW00000000.

## Electronic supplementary material

Supplementary Information(PDF 2291 kb)

Description of Additional Supplementary Files(PDF 161 kb)

Supplementary Data 1(XLSX 59 kb)

Supplementary Data 2(XLSX 50 kb)
