# Supplementary material for: Population genomics of finless porpoises reveal an incipient cetacean species adapted to freshwater
Source: Nat Commun. 2018 Apr 10;9:1276. doi: 10.1038/s41467-018-03722-x (PMC5893588; doi:10.1038/s41467-018-03722-x)
Supplement: Supplementary file 1 — Supplementary Information(PDF 2291 kb) [file 41467_2018_3722_MOESM1_ESM.pdf]

Supplementary Information for

Population genomics of finless porpoises reveal an incipient cetacean species adapted to freshwater

Xuming Zhou, Xuanmin Guang, Di Sun, Shixia Xu, Mingzhou Li, Inge Seim, Wencai Jie, Linfeng Yang, Qianhua Zhu, Jiabao Xu, Qiang Gao, Alaattin Kaya, Qianhui Dou, Bingyao Chen, Wenhua Ren, Shuaicheng Li, Kaiya Zhou, Vadim N Gladyshev, Rasmus Nielsen, Xiaodong Fang & Guang Yang

Table of content

Supplementary Figs. 1-13-------------------------------------Pages 2-10
Supplementary Tables 1-27---------------------------------Pages 11-38
Supplementary Notes 1-5------------------------------------Pages 39-47
References--Pages 48-53

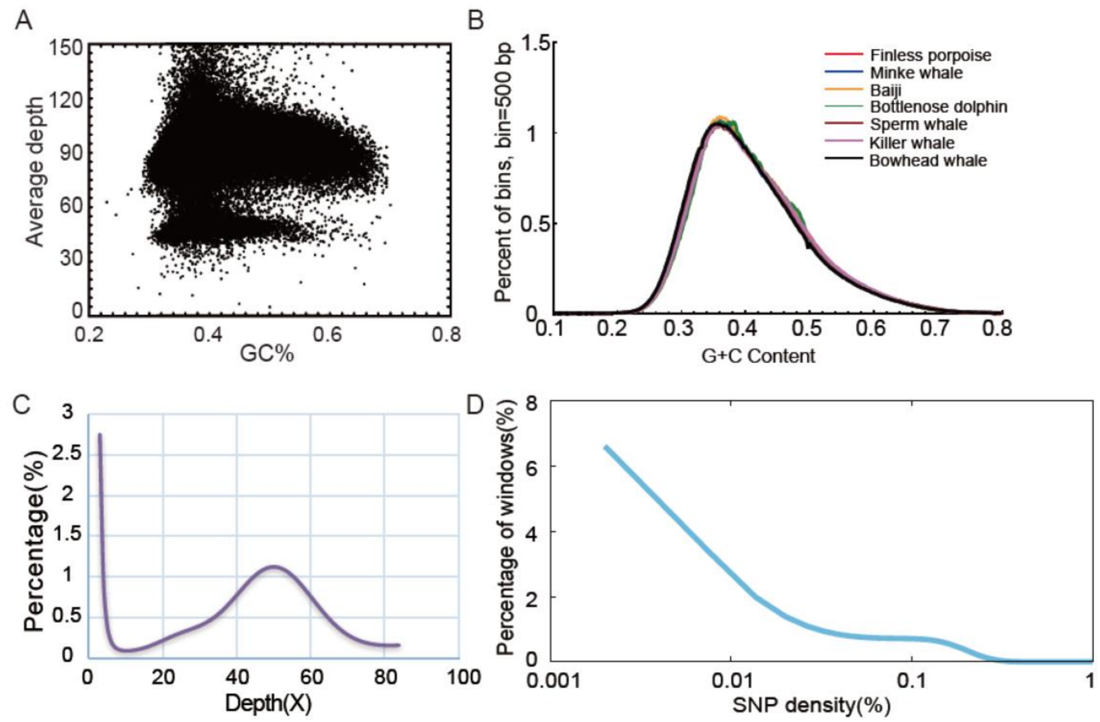

Supplementary Fig. 1. Sequencing and characteristic of finless porpoise genome assembly. (A) GC content against the sequencing depth of finless porpoise genome. Sliding windows of 10Kb without overlapping along the assembled sequence to calculate GC content and average sequencing depth. (B) G+C content distribution of seven whale genomes. Used sliding windows of 500bp with 250bp over-lapping to calculated each GC content. (C) 17-mer depth frequency distribution. Used 142.8Gb high-quality data to generate the 17-mer depth distribution curve frequency information. The peak depth is around 50 and estimated genome size is 2.49Gb. (D) Distribution of heterozygosity density. A total of 2.3M heterozygous SNPs were identified in the finless porpoise genome. Non-overlapping 50 kb windows were chosen and the heterozygosity density in each window was calculated. Note: finless porpoise: *Neophocaena asiaorientalis*, minke whale: *Balaenoptera acutorostrata*, baiji: *Lipotes vexillifer*, bottlenose dolphin: *Tursiops truncatus*, sperm whale: *Physeter catodon*, killer whale: *Orcinus orca*, bowhead whale: *Balaena mysticetus*.

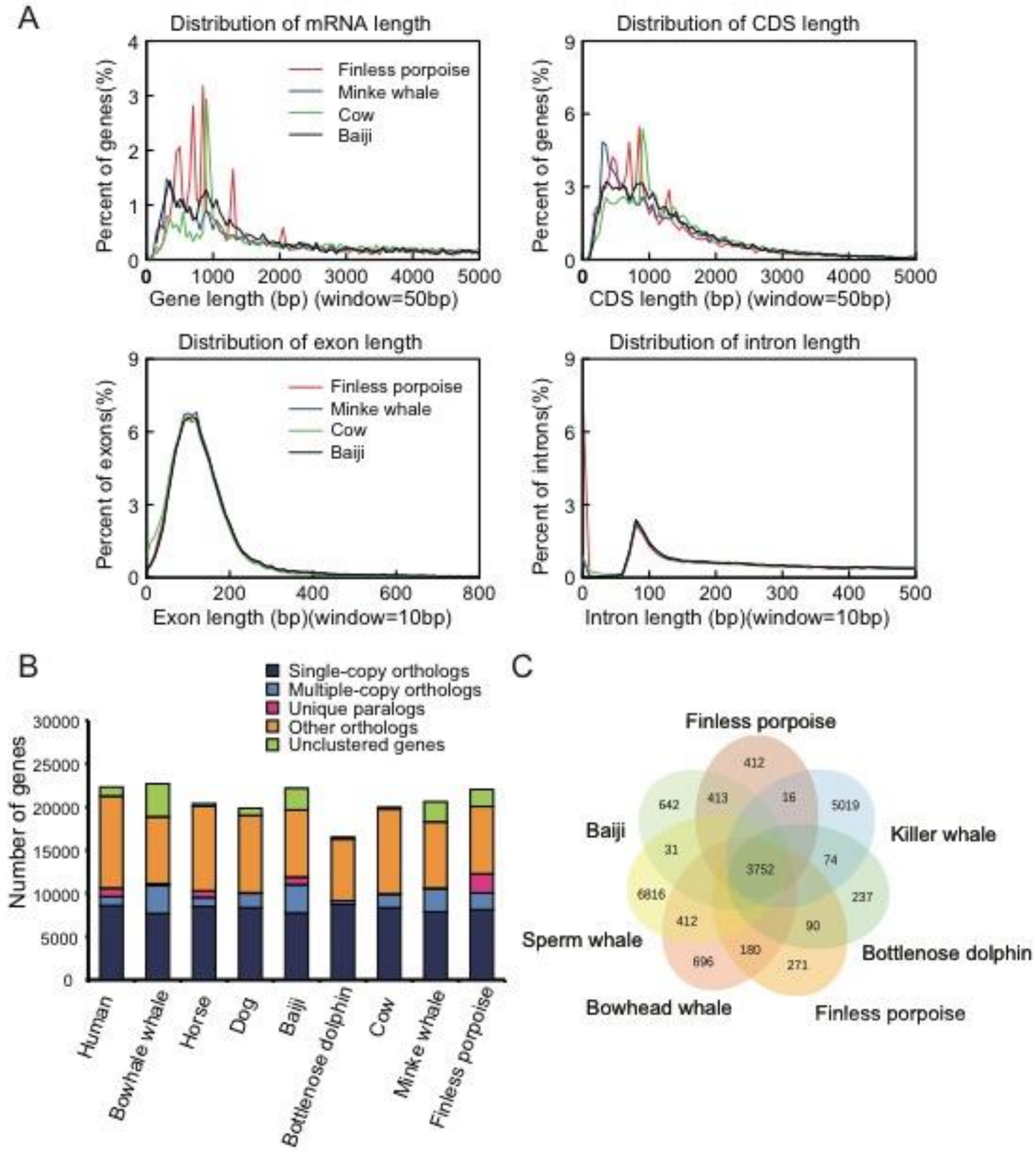

Supplementary Fig. 2. Characteristic of predicted protein-coding genes in finless porpoise genome. (A) Comparison of gene parameters among the finless porpoise and other three cetaceans. (B) Orthology delineation among the protein-coding gene family repertoires of the finless porpoise and other eight mammals. (C) Venn diagrams display the distribution of shared and unique gene families in seven sequenced whales. Note: human: *Homo sapiens*, cow: *Bos taurus*, dog: *Canis lupus* familiaris, horse: *Equus caballus*.

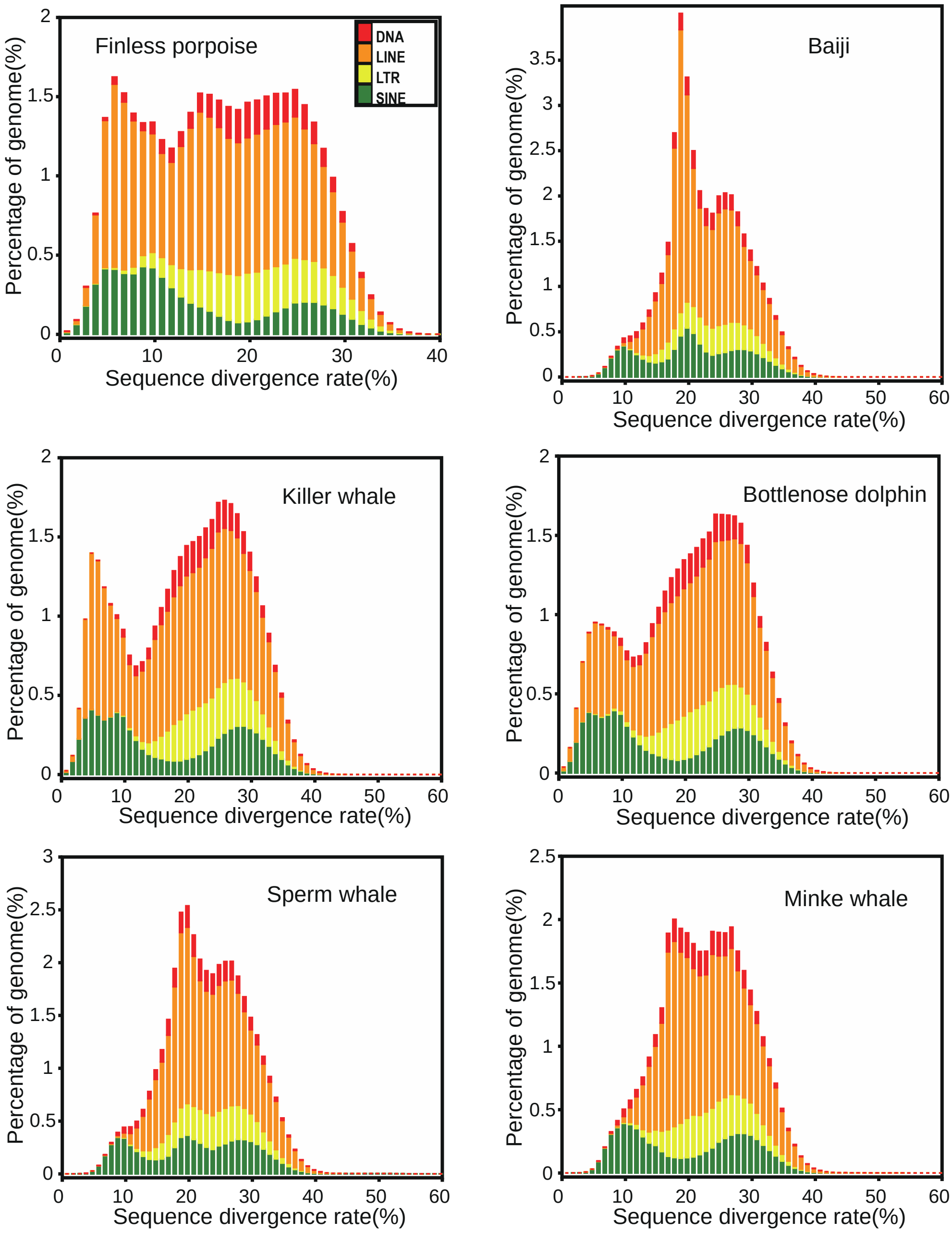

Supplementary Fig. 3. Divergence distribution of classified families of TEs in six cetacean genomes. The divergence rate was calculated based on the alignment between the RepeatMasker (http://www.repeatmasker.org) annotated repeat copies and the consensus sequence in the repeat library. The transposable elements comprise ~45.18% of the finless porpoise genome, which is similar to that of the common bottlenose dolphin (44%).

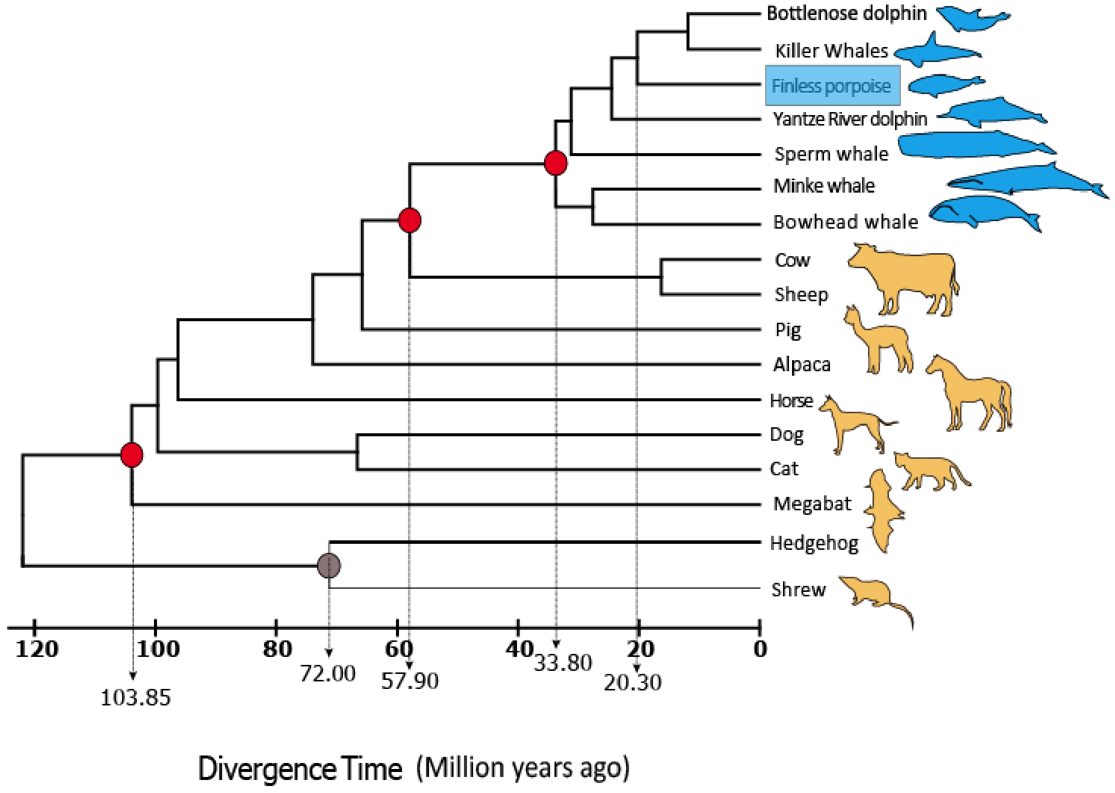

Supplementary Fig. 4. Phylogenetic tree and divergence times estimated for the finless porpoises and their relatives. The red solid circles on the branch nodes denote the node as an 'age constraint' used in the estimation of the time of divergence. The animal silhouette images were created by Xuming Zhou in Adobe Illustrator CS3.

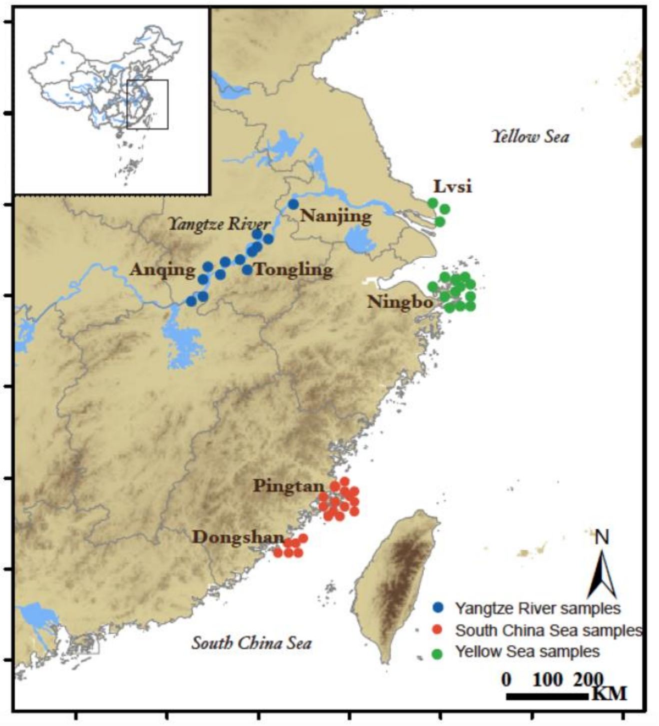

Supplementary Fig. 5. Schematic map showing finless porpoises sampled in this study, with sample size for each locality shown in supplementary Table 16. The map was retrieved from http://www.naturalearthdata.com (Public Domain; date accessed: Feb 2017) and generated using ArcGIS 9.3.

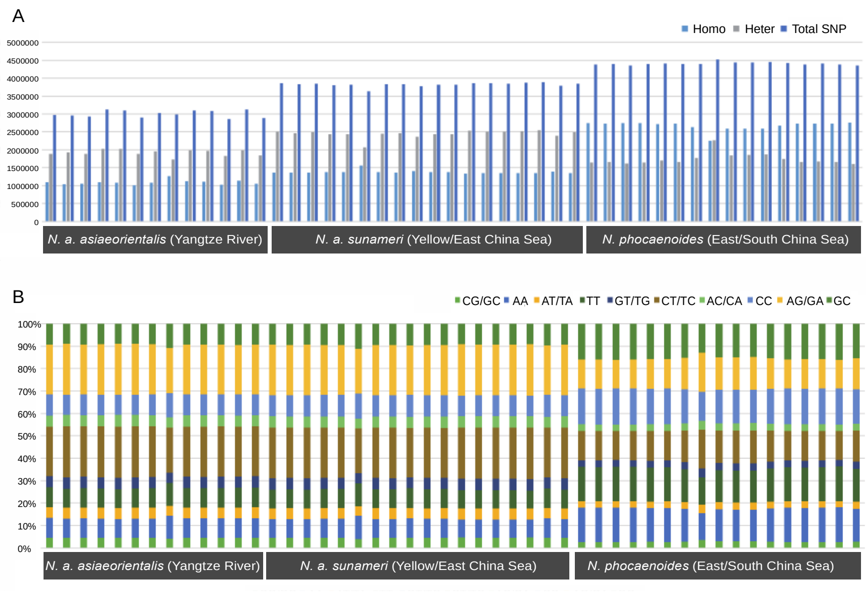

Supplementary Fig. 6. Characteristic of SNPs (single nucleotide polymorphisms) in finless porpoise genome. (A) Variant number for the 48 finless porpoise individuals at a population-scale. (B) Mutation spectrum for 48 finless porpoise individuals.

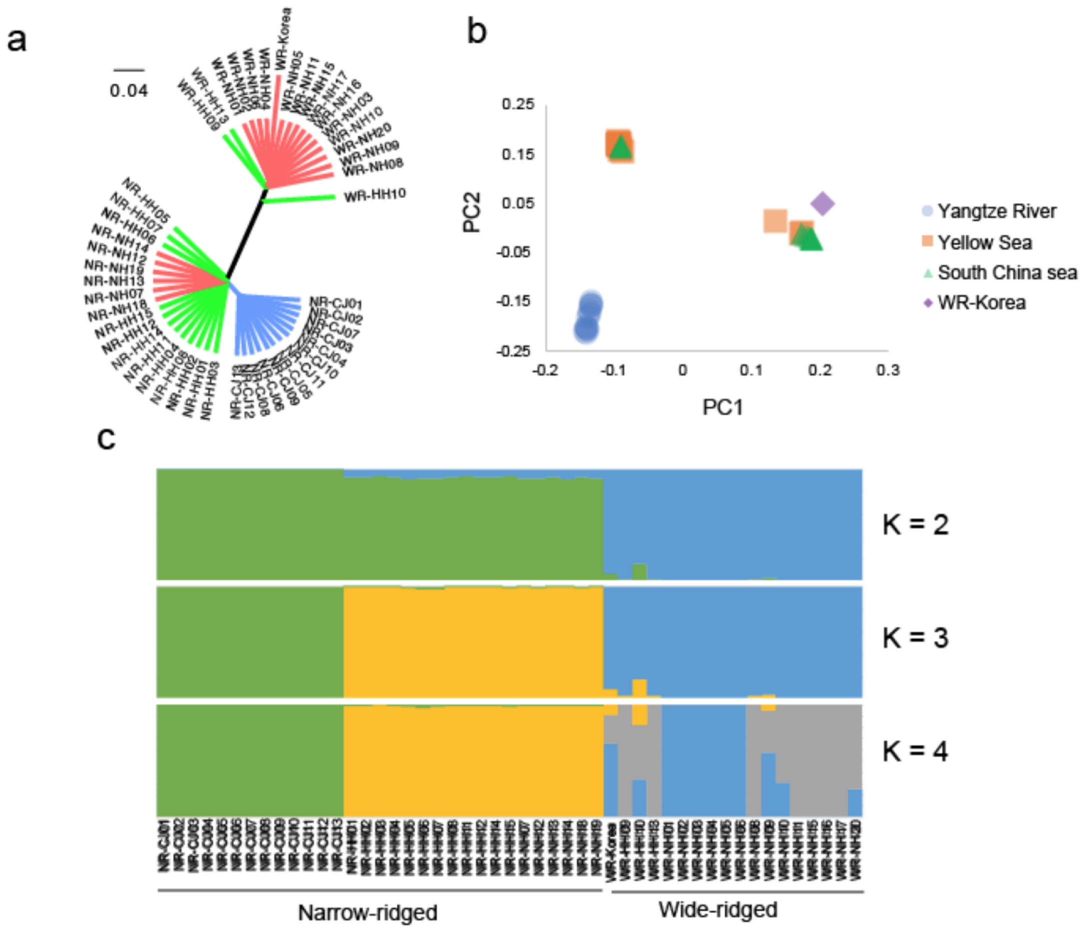

Supplementary Fig. 7. Phylogeny, principal components analysis and population structure of 48 finless porpoises and one additional individual reported previously (indicated as WR-Korea). (a) Neighbor-joining tree constructed from the allele-sharing matrix of variants of 49 finless porpoises. (b) Principal components analysis (PCA) of 49 finless porpoises. (c) Genetic structure of the 49 finless porpoises inferred by *frappe*, varying he number of admixture components (*K*) from 2 to 4.

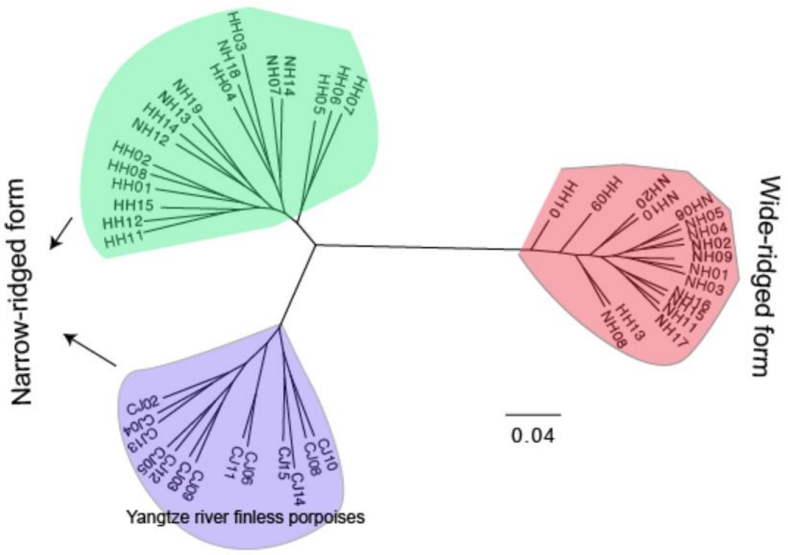

Supplementary Fig. 8. **Phylogenetic tree of 48 finless porpoises reconstructed using maximum likelihood method based on SNPs.**

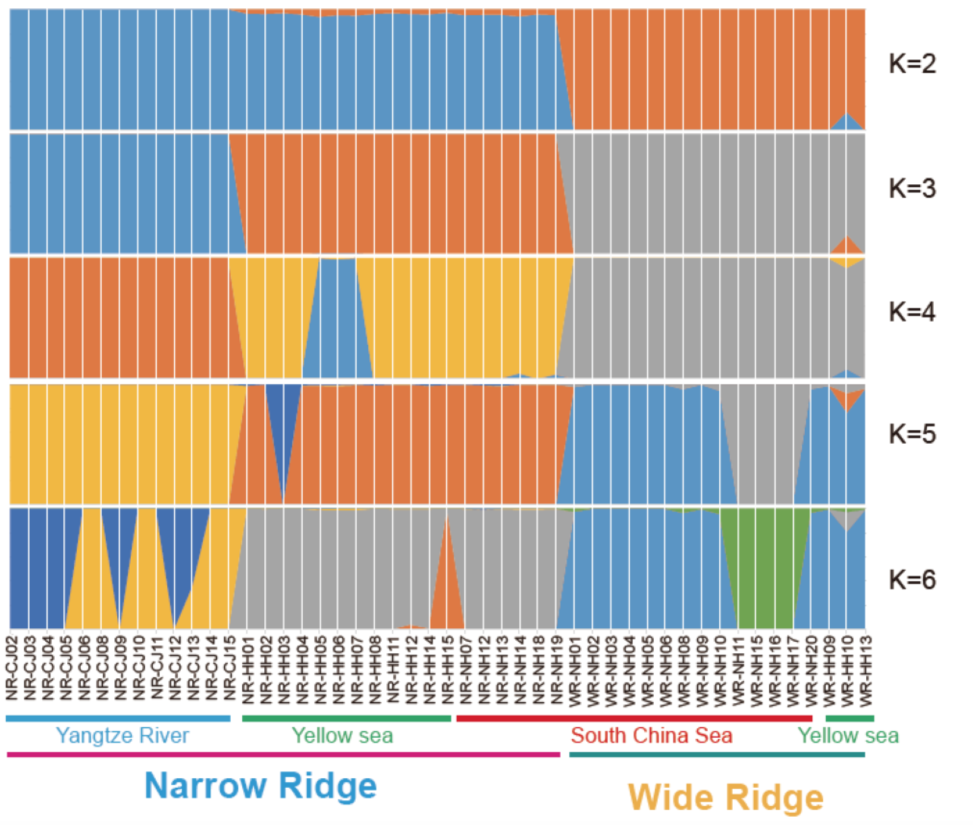

Supplementary Fig. 9. Genetic structure of the 48 finless porpoises inferred by *frappe*, varying he number of admixture components (*K*) from 2 to 5. The sample location for each individual is also indicated.

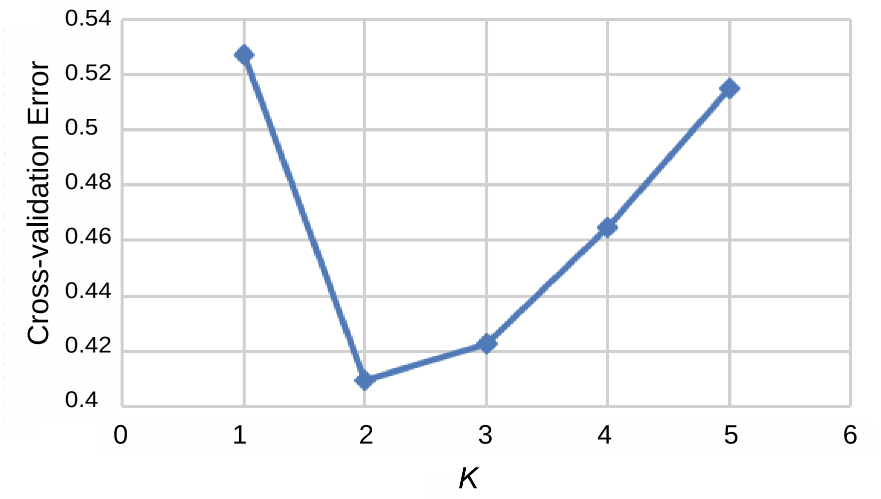

Supplementary Fig. 10. Cross-validation (CV) error for varying values of *K* in the ADMIXTURE analysis. Minimum of estimated CV error on *K*=2 or 3 suggests the most suitable number of ancestral populations.

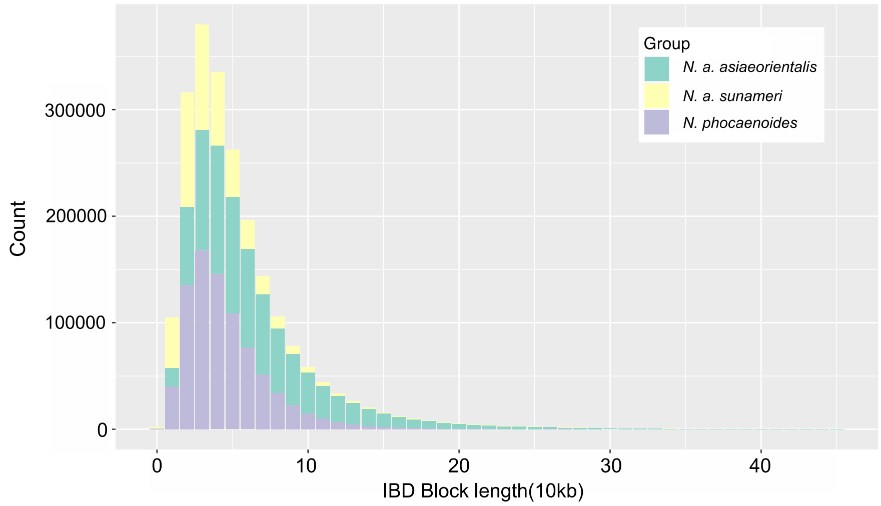

Supplementary Fig. 11. Distribution of IBD blocks length identified by BEAGLES in all three finless porpoise populations. Three finless porpoise populations are all showing relatively short (<100kb) IBD block length.

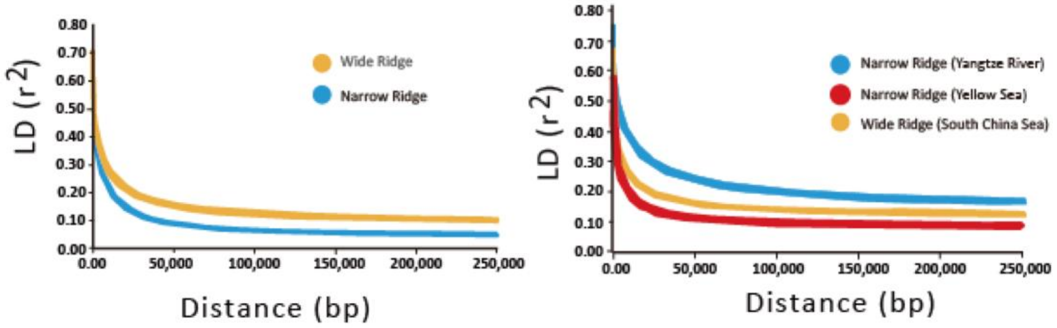

Supplementary Fig. 12. LD patterns for each of the two main forms (wide-ridged form and narrow-ridged form) and the three genetic clusters of finless porpoise inferred by phylogenetic trees.

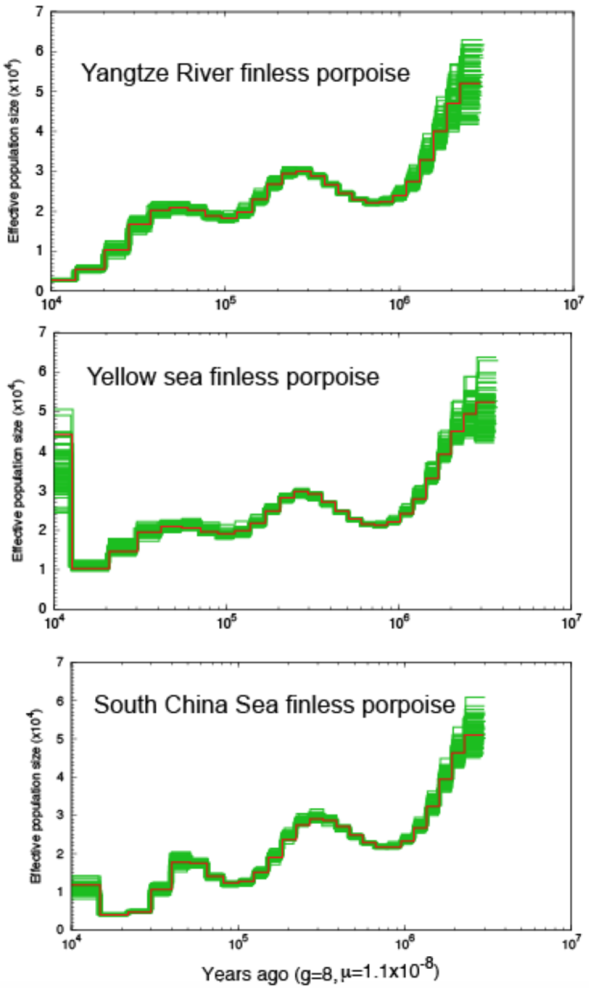

Supplementary Fig. 13. Demographic history of finless porpoises reconstructed from the reference and population resequencing genomes. The red line represents the estimated effective population size (N_e), and blue curves denote the 100 PSMC estimates resampled from the original sequence.

Supplementary Table 1. Summary of genome sequencing strategy for the finless porpoise.

Pair-end Libraries	Insert Size	Average Reads Length (bp)	Total raw Data (Gb)	Total clean Data (Gb)	Sequence Depth (×)	Physical Depth (×)
Solexa Reads	250bp	150	89.25	65.12	26.17	21.8
	500bp	150/100	92.14	77.6	31.2	63.8
	800bp	100	66.1	57.02	22.92	91.6
	2Kb	49	58.09	29.93	12.03	245.5
	5Kb	49	57.6	18.18	7.3	372.9
	10Kb	49	52.3	9.04	3.63	370.78
	20Kb	49	50.3	6.55	2.63	537.67
	40Kb	49	19.1	2.09	0.84	343.4
**Total**	/	/	484.88	265.53	106.72	2047.45

Note: All reads were calculated under the genome size of 2.49Gb.

Supplementary Table 2. Estimation of the finless porpoise genome size using K-mer analysis.

K	K-mernum	Peak depth	Genome Size	Used Bases	Used Reads	X
17	124,388,139,101	50	2,487,762,782	142,852,147,978	11,535,866,53	57.4

Note: The genome size is after error correction procedure that deleted 0.59% reads and 2.16% bases of sequencing errors.

Supplementary Table 3. Summary of finless porpoise genome assembly.

	Contig		Scaffold	
	**Size (bp)**	**Number**	**Size (bp)**	**Number**
**N90**	7,400	86,606	1,075,576	423
**N80**	12,237	63,034	2,344,531	281
**N70**	16,735	47,182	3,558,345	200
**N60**	21,472	35,181	4,936,175	145
**N50**	26,732	25,669	6,334,541	104
**Longest**	258,785	/	33,179,877	/
**Total Size**	2,278,605,083	/	2,295,152,199	/
**Total Number (>=100bp)**	/	264,651	/	97,387
**Total Number(>=2kb)**	/	127,772	/	2,179

Supplementary Table 4. Statistics of genome assembly of seven cetaceans.

	Contig N50		Scaffold N50		Total Size
	**Size (bp)**	**Number**	**Size (bp)**	**Number**	
Balaenoptera acutorostrata	22,571	31,010	12,843,668	57	2,442,893,294
Lipotes vexillifer	30,101	24,909	2,268,251	328	2,565,001,670
Balaena mysticetus	34,800	113,673	877	7,227	2,300,000,000
Physeter catodon	35,257	110,444	427,290	11,711	2,280,727,784
Tursiops truncatus	11,821	554,228	116,287	240,558	2,551,418,184
Neophocaena asiaeorientalis	26,732	25,669	6,334,541	104	2,295,152,199
Orcinus orca	70,300	80,100	12,735,091	1,668	2,372,919,875

Supplementary Table 5. RNA-seq mapping results of two finless porpoise blood sample.

Tissue	Read Types	Mapping to Finless Porpoise Genome	
		Number of reads	% of reads
Blood (sample 1)	Total reads	49,122,452	
	Mapped reads	34,293,894	69.81
	Multiple-|Uniquely-mapped reads	6,182,345|28,111,549	12.59|57.23
	Read-1|Read-2	17,213,827|17,080,067	35.04|34.77
	Non-splice reads| Splice reads	19,161,165|15,132,729	39.01|30.81
Blood (sample 2)	Total reads	67,519,566	
	Mapped reads	55,172,234	81.71
	Multiple-|Uniquely-mapped reads	12,453,047|42,719,187	18.44|63.27
	Read-1|Read-2	27,526,147|27,646,087	40.77|40.95
	Non-splice reads| Splice reads	42,437,338|12,734,896	62.85|18.86

Note: 'Splice reads' refers to reads where part of the read was not mapped contiguously to the reference genome.

Supplementary Table 6. Assessment of gene coverage by assembled finless porpoise transcripts.

Target Dataset	Number	Total length (bp)	Sequences covered by assembly (%)	With >90% sequence in one scaffold		With >50% sequence in one scaffold	
				Number	Percent	Number	Percent
**Finless porpoise**							
**>0**	72,056	54,592,774	98.71	6,916	95.98	71,685	99.48
**>200bp**	52,622	51,260,490	98.72	50,499	95.97	52,398	99.57
**>500bp**	24,600	42,881,568	98.69	23,332	94.84	24,521	99.68
**>1000bp**	15,021	36,122,263	98.63	14,107	93.9	14,967	99.64

Supplementary Table 7. Assessment of sequence coverage of the finless porpoise genome assembly using the CDS regions of the common bottlenose dolphin and baiji genomes.

Target Dataset	Number	Total length(bp)	Sequences covered by assembly (%)	With >90% sequence in one scaffold		With >50% sequence in one scaffold	
				Number	Percent	Number	Percent
**Baiji**							
**>0bp**	22,168	33,126,399	88.07	17,142	77.33	19,244	86.81
**>200bp**	22,022	33,101,208	88.00	17,026	77.31	19,101	86.74
**>500bp**	18,521	31,823,580	86.83	14,145	76.37	15,848	85.57
**>1000bp**	12,029	26,950,311	87.41	9,321	77.49	10,385	86.33
**Bottlenose dolphin**							
**>0bp**	16,611	28,583,459	99.84	12,391	74.60	16,443	98.99
**>200bp**	16,464	28,562,213	99.92	12,292	74.66	16,313	99.08
**>500bp**	14,770	27,930,577	99.95	10,906	73.84	14,644	99.15
**>1000bp**	10,703	24,874,202	99.97	7,752	72.43	10,622	99.24

Note: The CDS sequences of the common bottlenose dolphin and baiji were downloaded from NCBI, and mapped to the finless genome assembly. Out of 16,443 (86.81%) predicted protein-coding genes in the common bottlenose dolphin and 19,244 (86.81%) in baiji were covered by CDS regions of the finless porpoise genome.

Supplementary Table 8. Summary of predicted protein-coding genes in the finless porpoise genome compared with other representative mammalian genomes.

	Gene set	Number	Average transcript length (bp)	Average CDS length (bp)	Average exon per gene	Average exon length (bp)	Average intron length (bp)
De novo	AUGUSTUS	17,425	52,980	1,503	9.3	161	6,189
	GENSCAN	33,425	44,947	1,388	8.7	159	5,654
**Homolog**	*H. sapiens*	77,340	9,293	731	4.1	178	2,761
	T. truncates	23,913	22,121	1,310	7.5	174	3,179
	L. vexillifer	97,957	7,988	651	3.4	189	3,007
	B. acutorostrata	26,303	21,261	1,294	7.5	172	3,068
	C. familiaris	24,729	20,238	1,266	7.3	172	2,994
	S. scrofa	89,827	6,685	683	3.74	182	2,192
	B. taurus	26,481	19,374	1,251	7.12	175	2,993
**GLEAN**		14,160	35,522	1,616	9.4	171	4,032
**Final set**		22,014	25,175	1,260	7.2	175	3,864

Note: Genes with alternative splicing-induced premature termination and defective codon events were not considered.

Supplementary Table 9. Statistics of finless porpoise genes with functional classification by various methods.

		Number	Percent (%)
**Total**		22,014	/
**Annotated**	**InterPro**	14,950	67.91
	**GO**	11,834	53.76
	**KEGG**	14,182	64.42
	**Swissprot**	20,347	92.43
	**TrEMBL**	20,598	93.57
**Unannotated**		1,394	6.33

Note: Out of 22,014 predicted protein-coding genes in the finless genome, 20,620 (93.67%) have protein homologues in other mammalian genomes.

Supplementary Table 10. Summary of non-coding RNA genes in finless porpoise genome.

Type		Copy	Average length (bp)	Total length (bp)	% of genome
**miRNA**		506	84.3	42,664	0.0018
**tRNA**		231	76.7	17,732	0.00077
**rRNA**	**rRNA**	98	106.7	10,461	0.00046
	**18S**	22	114.86	2,527	0.0001
	**28S**	59	122.9	7,251	0.000316
	**5.8S**	1	95	95	0.000004
	**5S**	16	36.75	588	0.000026
	**snRNA**	576	113.93	65,626	0.0028
**snRNA**	**CD-box**	264	87.37	23,067	0.001
	**HACA-box**	206	136.8	28,184	0.001
	**splicing**	77	138.6	10,676	0.00047

Supplementary Table 11. Summary of transposon element families in finless porpoise genome based on various methods.

Type	Repeat Size (bp)	% of genome
TRF	37,426,548	1.63
Repeat Masker	871,794,971	37.98
Repeat Protein Mask	272,523,233	11.87
De novo	852,204,684	37.13
Total	1,036,969,371	45.18

Note: Transposable elements comprised ~45.18 % of the finless porpoise genome, which is similar to the value obtained for other cetacean genomes (Baiji: 43.2%, Bowhead whale: 41.0%, Minke whale: 37.34%).

Supplementary Table 12. Statistics of classified repeat of finless porpoise genome.

	RepBase TEs		TE Proteins		De novo		Combined TEs*	
	Length (bp)	%in Genome	Length (bp)	% in Genome	Length (bp)	% in Genome	Length (bp)	% in Genome
**DNA**	73,495,749	3.2	8,285,071	0.36	6,468,260	0.28	78,269,225	3.4
**LINE**	524,943,063	22.87	254,706,668	11.1	738,365,766	32.17	870,140,323	37.9
**LTR**	125,700,121	5.48	9,560,292	0.42	124,591,106	5.43	238,878,903	10.4
**SINE**	152,428,634	6.64	/	/	48,216,839	2.1	186,500,639	8.1
**Other†**	613	0.00003	/	/	/	/	613	/
**Unknown‡**	/	/	/	/	10,153,713	0.44	10,153,713	0.44
**Total**	871,794,971	37.98	272,523,233	11.87	851,395,063	37.1	1,024,994,072	44.66

Note: *Combined: the non-redundant consensus of all repeat prediction/classification methods employed. †Other: the repeats classified by RepeatMasker, which are not included in the other groups; ‡Unknown: the predicted repeats that cannot be classified by RepeatMasker; LINE, long interspersed nuclear elements; LTR, long terminal repeat; SINE, short interspersed nuclear elements.

Supplementary Table 13. Summary of syntenic regions among the finless porpoise, Baiji, minke whale and cow genomes. The statistics were counted based on chromosomes in cow genome.

Species vs Species	Aligned Length(bp)	Target Genome Coverage Rate	Query Genome Coverage Rate
Baiji *vs* Finless porpoise	1,860,137,553	91.3%	81.0%
Minke whale *vs* Finless porpoise	1,996,336,120	97.5%	86.98%
*Cow *vs* Finless porpoise	2,048,936,051	96.3	89.3%

Note: Coverage rate of Cow *vs* Finless porpoise is relatively higher, probably effected by that the cow genome is a chromosome assembly.

Supplementary Table 14. List of positively selected genes identified in finless porpoises using branch site model.

Gene ID	Parameters (Null)	LnL (Null)	Parameters (Alternative)	LnL (Alternative)	P (LRT)
STT3A	36	-3344.92	37	-3332.26	4.87E-07
KIAA1024L	36	-1170.43	37	-1165.35	1.44E-03
GPAM	36	-2349.78	37	-2331.32	1.23E-09
HIPK1	36	-5891.63	37	-5886.33	1.13E-03
TMOD1	36	-2025.34	37	-1993.31	1.20E-15
FGL2	36	-4975.53	37	-4973.59	4.90E-02
RPF1	36	-3529.60	37	-3519.90	1.05E-05
HSD17B6	36	-2157.25	37	-2150.88	3.57E-04
TXNL1	36	-977.54	37	-967.72	9.33E-06
PTPRZ1	36	-8279.00	37	-8257.51	5.54E-11
MMP13	36	-542.93	37	-540.72	3.54E-02
CPNE3	36	-2993.03	37	-2983.73	1.60E-05
ESPL1	36	-11413.14	37	-11411.05	4.11E-02
TYRP1	36	-3000.96	37	-2995.96	1.57E-03
NRG3	36	-1492.18	37	-1486.17	5.30E-04
COMMD6	36	-800.62	37	-794.71	5.83E-04
AP5M1	36	-959.45	37	-956.10	9.62E-03
DST	36	-8559.25	37	-8545.89	2.35E-07
SARAF	36	-3843.47	37	-3829.15	8.66E-08
CYP2R1	36	-2946.61	37	-2942.65	4.91E-03
CHN2	36	-1964.50	37	-1962.46	4.32E-02
CD3E	36	-2940.79	37	-2938.56	3.47E-02
DONSON	36	-2867.89	37	-2857.72	6.49E-06
FAM214A	36	-5373.96	37	-5369.65	3.32E-03
AHI1	36	-1933.61	37	-1927.03	2.86E-04
PLD1	36	-3326.29	37	-3314.54	1.25E-06
LMAN1	36	-3246.56	37	-3225.83	1.20E-10

MPHOSPH6	36	-1124.10	37	-1115.92	5.27E-05
SEL1L3	36	-2367.54	37	-2361.17	3.59E-04
GABPB2	36	-3858.03	37	-3830.26	9.14E-14
PDE7A	36	-875.01	37	-867.35	9.14E-05
RNF144A	36	-799.81	37	-797.05	1.88E-02
TTLL6	36	-1437.58	37	-1435.39	3.65E-02
NPY	36	-726.01	37	-721.50	2.67E-03
FANCD2	36	-10500.95	37	-10498.77	3.67E-02
LYST	36	-22154.85	37	-22148.81	5.06E-04
DCK	36	-705.99	37	-697.00	2.24E-05
PTPRC	36	-5265.00	37	-5261.18	5.71E-03
TMEM107	36	-2030.46	37	-2005.35	1.37E-12
C11orf57	36	-1963.97	37	-1961.60	2.95E-02
NFYC	36	-952.82	37	-944.23	3.42E-05
TTBK2	36	-8204.08	37	-8169.04	5.67E-17
VWA5B2	36	-6569.08	37	-6566.35	1.95E-02
SSMEM1	36	-2044.41	37	-2038.21	4.28E-04
LYPD6B	36	-1059.60	37	-1057.08	2.48E-02
CRY1	36	-1534.91	37	-1529.34	8.42E-04
CC2D2A	36	-1801.75	37	-1786.92	5.16E-08
TPH1	36	-1678.40	37	-1673.27	1.35E-03
RB1CC1	36	-4165.54	37	-4135.59	9.94E-15
NRD1	36	-2644.25	37	-2641.09	1.20E-02
TOX4	36	-4993.15	37	-4954.02	9.03E-19
IGSF11	36	-3184.65	37	-3182.19	2.67E-02
UBA5	36	-1533.42	37	-1529.08	3.23E-03
TLR3	36	-11398.61	37	-11374.46	3.63E-12
BAIAP2L1	36	-2880.35	37	-2873.55	2.27E-04
DEPDC7	36	-4873.19	37	-4858.47	5.75E-08
MCMDC2	36	-3143.53	37	-3132.43	2.48E-06

Supplementary Table 15. Information of three subspecies of finless porpoises.

	Narrow ridge form		Wide-ridge form
**Classification and nomenclature**	*N. a. asiaeorientalis*<br>Yangtze finless porpoise	*N. a. sunameri*<br>East Asian finless porpoise	*N. phocaenoides*<br>Indo-Pacific finless porpoise
**Distribution**	In the Yangtze River (up to 1600 km inland) and in some associated lakes and estuaries (Poyang and Dongting lakes; Gan Jiang and Xiang Jiang rivers)	Northern part of East China Sea, the Yellow/Bohai Seas, and the waters of Korea and Japan (potentially sympatric with *N. p. phocaenoides* in the Taiwan Strait).	Southern part of East China Sea, the South China Sea, mainland coast of southern Asia west to the Persian Gulf.
**Narrow/Wide-ridge**	A similar dorsal surface but less tubercules compare with *N. a. sunameri*.	A narrow tuberculed area on the dorsal ridge.	A wide area of tubercules and more than 10 rows of denticles on the dorsal surface.
**Habitat**	Fresh water	Sea water	Sea water

Supplementary Table 16. Sequencing information of 48 finless porpoise samples.

Sample ID*	Locality	Sample location	Wild/Narrow ridge	Type of samples	Raw data			Clean data		
					Total bases (Gb)	GC percent (%)	Q20 percent (%)	Total bases (Gb)	GC percent (%)	Q20 percent (%)
NR_CJ01	Anqing, Anhui	Yangtze River	Narrow	Muscle	37.77	41.37	90.11	34.59	41.47	93.96
NR_CJ02	Anqing, Anhui	Yangtze River	Narrow	Muscle	34.79	42.39	94.32	33.09	41.99	96.39
NR_CJ03	Nanjing, Jiangsu	Yangtze River	Narrow	Muscle	36.80	42.24	93.94	35.12	42.15	95.99
NR_CJ04	Xiaogu Mountain, Anqing, Anhui	Yangtze River	Narrow	Muscle	37.90	42.24	93.39	36.14	42.15	95.47
NR_CJ05	Wangjiang county, Anqing, Anhui	Yangtze River	Narrow	Muscle	38.36	41.73	92.90	36.37	41.60	95.19
NR_CJ06	Xijiang, Anqing, Anhui	Yangtze River	Narrow	Muscle	103.43	42.77	93.34	98.05	42.59	95.47
NR_CJ07	Zongyang, Anqing, Anhui	Yangtze River	Narrow	Muscle	35.93	42.20	90.69	33.07	42.01	94.18
NR_CJ08	Tongling, Anhui	Yangtze River	Narrow	Blood	30.19	41.59	95.31	29.28	41.49	96.55
NR_CJ09	Tongling, Anhui	Yangtze River	Narrow	Blood	34.81	41.21	94.96	33.65	41.08	96.36
NR_CJ10	Tongling, Anhui	Yangtze River	Narrow	Blood	34.15	41.44	94.13	32.75	41.34	95.88
NR_CJ11	Tongling, Anhui	Yangtze River	Narrow	Blood	34.66	41.07	93.98	33.17	40.92	95.76
NR_CJ12	Tongling, Anhui	Yangtze River	Narrow	Blood	38.10	40.99	92.79	36.40	40.80	94.66

NR-CJ13	Tongling, Anhui	Yangtze River	Narrow	Blood	37.87	40.94	92.89	36.16	40.72	94.79
NR-HH01	Ningbo, Zhejiang	East China Sea	Narrow	Muscle	37.07	42.69	94.89	35.59	42.54	96.61
NR-HH02	Ningbo, Zhejiang	East China Sea	Narrow	Muscle	34.64	42.40	93.55	32.89	42.04	95.65
NR-HH03	Ningbo, Zhejiang	East China Sea	Narrow	Muscle	35.83	41.90	91.45	31.32	41.43	95.25
NR-HH04	Ningbo, Zhejiang	East China Sea	Narrow	Muscle	36.18	41.90	93.40	32.57	41.55	95.90
NR-HH05	Lvsi, Jiangsu	Yellow Sea	Narrow	Muscle	37.20	42.64	94.71	35.68	42.46	96.49
NR-HH06	Lvsi, Jiangsu	Yellow Sea	Narrow	Muscle	35.68	42.45	94.05	31.77	42.05	92.70
NR-HH07	Lvsi, Jiangsu	Yellow Sea	Narrow	Muscle	35.50	42.71	93.90	33.64	42.26	96.13
NR-HH08	Ningbo, Zhejiang	East China Sea	Narrow	Muscle	34.24	42.72	94.60	32.91	42.67	96.35
WR-HH09	Ningbo, Zhejiang	East China Sea	Wide	Muscle	38.30	41.44	92.59	36.18	41.31	94.91
WR-HH10	Ningbo, Zhejiang	East China Sea	Wide	Muscle	34.09	41.86	95.53	33.03	41.74	96.85
NR-HH11	Ningbo, Zhejiang	East China Sea	Narrow	Muscle	29.12	43.21	95.52	28.09	43.17	97.06
NR-HH12	Ningbo, Zhejiang	East China Sea	Narrow	Muscle	30.44	43.42	95.18	29.34	43.40	96.81
WR-HH13	Ningbo, Zhejiang	Yellow Sea	Wide	Muscle	39.04	42.62	93.77	37.33	42.40	95.46
NR-HH14	Ningbo, Zhejiang	Yellow Sea	Narrow	Muscle	32.14	43.26	95.13	30.97	43.23	96.75
NR-HH15	Ningbo, Zhejiang	Yellow Sea	Narrow	Muscle	100.79	42.69	91.13	94.02	42.46	94.08
WR-NH01	Dongshan, Fujian	South China Sea	Wide	Muscle	36.42	42.48	93.28	34.75	42.32	95.31
WR-NH02	Dongshan, Fujian	South China Sea	Wide	Muscle	36.32	43.21	93.49	34.61	43.03	95.42
WR-NH03	Dongshan, Fujian	South China Sea	Wide	Muscle	24.97	42.97	96.02	24.14	42.89	97.33
WR-NH04	Dongshan, Fujian	South China Sea	Wide	Muscle	35.75	42.58	93.92	34.27	42.45	95.77
WR-NH05	Dongshan, Fujian	South China Sea	Wide	Muscle	36.57	42.61	93.17	34.90	42.45	95.20
WR-NH06	Dongshan, Fujian	South China Sea	Wide	Muscle	36.44	42.88	93.83	34.88	42.74	95.60
NR-NH07	Pingtan, Fujian	South China Sea	Narrow	Muscle	37.39	42.47	94.00	35.78	42.30	95.86

WR-NH08	Pingtan, Fujian	South China Sea	Wide	Muscle	106.60	42.44	93.36	101.58	42.22	95.39
WR-NH09	Pingtan, Fujian	South China Sea	Wide	Muscle	32.56	41.47	95.94	31.55	41.34	97.22
WR-NH10	Pingtan, Fujian	South China Sea	Wide	Muscle	38.36	41.19	94.64	36.60	40.99	96.19
WR-NH11	Pingtan, Fujian	South China Sea	Wide	Muscle	38.83	42.05	94.63	35.91	41.74	96.19
NR-NH12	Pingtan, Fujian	South China Sea	Narrow	Muscle	36.58	42.26	92.48	34.76	42.10	94.71
NR-NH13	Pingtan, Fujian	South China Sea	Narrow	Muscle	71.20	42.62	92.18	66.53	42.37	94.93
NR-NH14	Pingtan, Fujian	South China Sea	Narrow	Muscle	72.93	42.40	91.41	68.09	42.13	94.21
WR-NH15	Pingtan, Fujian	South China Sea	Wide	Muscle	30.78	42.37	95.21	29.81	42.25	96.52
WR-NH16	Pingtan, Fujian	South China Sea	Wide	Muscle	38.51	42.90	94.09	36.71	42.68	95.73
WR-NH17	Pingtan, Fujian	South China Sea	Wide	Muscle	32.91	44.92	94.99	31.61	44.84	96.77
NR-NH18	Pingtan, Fujian	South China Sea	Narrow	Muscle	27.62	44.49	96.18	26.78	44.43	97.55
NR-NH19	Pingtan, Fujian	South China Sea	Narrow	Muscle	36.06	42.25	90.97	33.70	42.01	93.89
WR-NH20	Pingtan, Fujian	South China Sea	Wide	Muscle	36.88	41.85	91.86	34.97	41.66	94.04

Note: Sample ID composed of ridge form and sample location: WR: Wide ridge form, NR: Narrow ridge form. CJ: Yangtze river, HH: Yellow sea, NH: South China sea.

Supplementary Table 17. Summary of SNP calling for three finless porpoise populations.

Category	Narrow ridge form Yangtze River samples	Narrow ridge form Marine samples	Wide ridge form samples
Sample Size	13	15	20
Number of total SNPs	6,412,544	11,016,939	10,389,807
Number of shared SNPs		5,187,414	

Supplementary Table 18. Summary of SNPs annotation in three species/populations of finless porpoises.

	Category	Narrow ridge form Yangtze River samples	Narrow ridge form Marine samples	Wide ridge form samples	All samples
**INTERGENIC**	Intergenic region	4,580,162	7,429,137	7,397,933	9,460,709
**UPSTREAM**	Upstream gene variant	271,643	447,164	446,242	576,336
**EXONIC**	Initiator codon variant	7	9	9	13
	Start lost	85	123	120	151
	Missense variant	23,606	41,156	41,503	56,770
	Stop gained	538	1,053	1,014	1,464
	Stop lost	48	67	64	77
	Stop retained variant	12	30	28	40
	Synonymous variant	23,508	39,960	40,045	52,913
**INTRONIC**	Intragenic variant	10,092	16,767	17,152	21,821
	Intron variant	1,233,293	2,015,366	2,012,296	2,590,324
	Splice acceptor variant	169	260	286	344
	Splice donor variant	143	223	221	287
	Splice region variant	3,271	5,440	5,487	7,267
**DOWNSTREAM**	Downstream gene variant	237,344	390,876	390,772	504,491

Supplementary Table 19. Tracy-Widom (TW) statistics for the first four eigenvalues from PCA analysis of finless porpoise.

Eigenvectors	Eigen-value	Tw-state	p-value
1	24.574715	14.095	3.47E-17
2	5.798835	26.417	3.04E-41
3	2.533162	-4.901	0.999593
4	2.470107	-5.498	0.999967

Supplementary Table 20. **Summary of *f3-statistic*.**

Source 1 (A)	Source 2 (B)	Target (C)	f3 mean	std. err	Z
Yangtze River samples	Narrow-ridge marine samples	Wide-ridge formed samples	0.009443	0.000226	41.762
Yangtze River samples	Wide-ridge formed samples	Narrow-ridge marine samples	-0.007938	0.000185	-43.014
Narrow-ridge marine samples	Wide-ridge formed samples	Yangtze Rive samples	0.059055	0.000477	123.709

Note: If C is significantly admixed (Z-score < 3), then f3 (C; A, B) has a negative mean. Then A and B contributed to the genome of the admixed strain. If f3 is positive, it does not mean strains are not admixed. f3-statistic is influenced by population specific drift.

Supplementary Table 21. **Path sampling results for species delimitation analyses.** Bayes factor (BF) calculations are made against the three-species model (Run A). A positive BF values indicate support for the three-species model and BF > 10 is decisive.

Model	Species	MLE	BF
Run A, three species model	3	-5.7×10^6	
Run B, two species model	2	-6.9×10^6	$>10^6$

Note: MLE = Marginal likelihood estimate.

Supplementary Table 22. A list of sweep regions, using an outlier approach in wide-ridged populations. Top 19 peaks with CLR values larger than the genome wide 99.8% quantile are shown. Consecutive outlier CLR values are merged to a single sweep region.

Rank	Chromosome	Position (Mbp)	Max CLR	Genes associated with peak	Gene closest to peak	Distance between peak and closest gene (Mbp)
1	8	9.1	882.62	*ATG10, Atf4, RPS19BP1, Mchr1, Cacna1i, Rpl23a, ADSL, COX6A1, PCDH20, FAM83F, smcr7l, IMP3, MKL1, ATP6AP1L, GRAP2, Tnrc6b, ENTHD1*	*Rpl23a*	0.16
2	17	53.2	597.89	*WDR64, EAN57, TST, MPST, FOXRED2, EXO1, BECN1L1, CEP170, PVALB, Txn2, EIF3D, NCF4, CSF2RB, FH, IFT27, KMO*	*BECN1L1*	0.36
3	18	68.7	529.95	*KDM6A, LRRC69, SLC26A7, RUNX1T1, Otud6b, TMEM55A, VCAM1, TMEM64, NECAB1*	*SLC26A7*	0.05
4	13	45.1	519.23	*GPCPD1, TRMT6, CHGB, RPL29, KCNMA1, PCNA, MCM8, RASSF2, PROKR2, FERMT1, LRRN4, PRNT, PDXK, SLC23A2, PRND*	*PROKR2*	0.09
5	13	62.5	491.89	*DDI1, DCUN1D5, Gria4, THY1, USP2, GPR45, KBTBD3, CASP13, RNF26, DYNC2H1, MFRP, MMP13, MCAM*	*CASP13*	0.35
6	9	73.2	377.16	*DIMT1L, RPL21, LSMD1, ZPLD1, ENOPH1, IPO11*	*ENOPH1*	0.33
7	3	38.8	374.19	*BPGM, FPGT, LRRIQ3*	*LRRIQ3*	0.65
8	15	48.2	362.21	*CYP27C1, BIN1, GYPC, POU2F1, ERCC3, PROC, DUSP27, MAP3K2*	*GYPC*	0.32
9	14	43.8	357.73	*CAPN8, CAPN2, TLR5, TP53BP2, C1orf65, Susd4*	*TP53BP2*	0.49

10	10	67.9	352.75	*DUSP19, SSFA2, PPP1R1C, nckap1, NUP35, DNAJC10, pro-pol, FRZB*	*B0S6R1*	0.03
11	2	78.2	354.87	*PNPLA2, ODF3, ETV4, LRDD, Usp6nl, LSM12, HDAC5, Mpp2, IFITM5, UBTF, CDHR5, Taldo1, MUC6, Mob2, B4GALNT4, TSPAN4, BRSK2, ATP6V0E1, TMEM80, DRD4, Dusp8, SYT8, C11orf89, Ano9, Pkp3, IRF7, RPLP2, G6PC3, PSMD13, NAGS, PDDC1, SIRT3, Arl4d, RNH1, EFCAB4A, H-RAS, MEOX1, RIC8A, Deaf1, PTDSS2, CD300LG, PYY, DHX8, Sost, MUC5B, TOLLIP, CHID1, C11orf35, AP2A2, PPY, Cend1, MUC5AC, MPP3, IFITM1, IFITM3, PHRF1, ASB16, TMEM101, C17orf53, MUC2, ATHL1, LRRC56, Tnnt3, BET1L, LSP1, CTSD*	*Deaf1*	0.008
12	3	14.1	351.74	*RHAG, OPN5, CD2AP, C6orf138, C6orf138, pro-pol, TFAP2B, C6orf138, C6orf141, TFAP2D, MUT*	*MUT*	0.01
13	21	63.6	340.94	*EI24, SLITRK4, SLC25A32, RPS3A, CTHRC1, DCAF13, ASB3, TSPAN7, ERLEC1, PSME4, POF1B*	*TSPAN7*	0.41
14	20	56.2	339.75	*MAGI1, Tfap2a, UBLCP1, OFCC1, MAGI1, Prim2, Magi1, FTL, GCNT2*	*UBLCP1*	0.027
15	7	28.8	302.38	*AIPL1, Ankfy1, ASPA, ATP2A3, C17orf100, CAMKK1, CCDC92, CTNS, CYB5D2, FAM64A, FBXO39, ITGAE, KIAA0664, KIAA0753, LLGL1, Mettl16, MYBBP1A, P2rx5, Pafah1b1, PITPNM3, Rap1gap2, Rpl10a, SGSM2, Shpk, SLC13A5, SPATA22, SPNS3, SRR, TAX1BP3, TEKT1, Tmem93, TRPV1, TSR1, TXNDC17, Ube2g1, WSCD1, ZZEF1*	*ZZEF1*	0.033
16	12	55.3	269.89	*Rhob, APOB, GDF7, HS1BP3, Tdrd6*	*Tdrd6*	0.37
17	18	31.4	245.38	*SH3BP5, AMOT, LHFPL1*	*AMOT*	0.29
18	16	57.7	236.78	*Ttdn1, TTC18, C10orf103, C7orf10, PLAU, Cdk13, NDST2, Ppp3cb, CAMK2G, USP54, SEC24C, DNAJC9, VCL, SYNPO2L, KIAA0913*	*Ttdn1*	0.04
19	8	106.7	232.62	*Lrfn5*	*Lrfn5*	0.05

Supplementary Table 23. A list of sweep regions, using an outlier approach in narrow-ridged populations. Only top 20 regions with CLR values larger than the genome wide 99.8% quantile are shown. Consecutive outlier CLR values are merged to a single sweep region.

Rank	Chromosome	Position (Mbp)	Max CLR	Gene with peak	Gene closest to peak	Distance between peak and closest gene (Mbp)
1	8	31.9	507.42	*CTAGE5*	*CTAGE5*	0.89
2	15	10.5	243.43	*Mapk10, ARHGAP24, CDS1, DUSP11, WDFY3*	*ARHGAP24*	0.34
3	9	66.9	219.43	*GTF2B*	*GTF2B*	0.06
4	17	23	183.71	*RPL17, SYNCRIP, SNX14, Rps6, TBX18, 5HT1E, Smek1, RPS2, NT5E, MRAP2, KIAA1009*	*RPL17*	0.16
5	2	52.5	182.56	*SETMAR, TEAD1, Rps28, KIAA0895*	*KIAA0895*	0.32
6	10	85.6	168.92	*BDP1, DACH1.*	*BDP1*	0.34
7	20	49.4	161.53	*INPP1, OAZ2, PPP2R2B, CALML4, PIAS1, SKOR1*	*INPP1*	0.14
8	4	96.6	160	*HS3ST1, Rab28, BOD1L, NKX3-2*	*HS3ST1*	0.86
9	11	33.3	158.79	*SHISA6, Elac2, C2orf67, ZNF18, Arhgap44, PIRT, MAP2K4, DNAH9, SHISA6*	*ZNF18*	0.04
10	2	94.7	155.51	*Txnl4a, TIGD1, PQLC1, Ctdp1, KCNG2, RBFA, ADNP2, NFATC1, RNF113A, TTC17*	*ADNP2*	0.35
11	5	1.4	149.89	*SCN3A, Scn1a, GALNT3, COBLL1, SLC38A11, HPRT1, SCN9A, SCN7A, CSRNP3, GRB14, TTC21B, RPS4*	*RPS4*	0.046
12	14	87.3	149.34	*SETMAR*	*SETMAR*	1.00

13	7	63.2	142.59	*Ptbp2, TMEM56, PHKB*	*Ptbp2*	0.33
14	17	89.6	140.33	*Cdh12, CHRDL1, VPS29*	*Cdh12*	0.23
15	14	8.2	136.16	*EYS, PHF3, MLL3, GALNT11*	EYS	0.18
16	18	48.2	125.61	*C3orf32, SETD5, SRGAP3, CAV3, SRGAP3, TIGD1, RPL37A, LHFPL4, THUMPD3, RAD18, LMCD1, OXTR*	*RAD18*	0.036
17	13	85.8	115.65	*MTO1, FILIP1, OOEP, CD109, EEF1A1, TMEM30A, MB21D1, COL12A1, ECAT1, COX7A2, DDX43, SLC17A5, KHDC1*	*CD109*	0.39
18	19	71.9	113.12	*TRH, VPS35, MYLK3, GRIP2, QtsA-20224, C3orf20, C3orf19, Rpl32, ORC6, SHCBP1, IFT122, RHO, SET, PLXND1, MRPS25, HMGN1, FGD5, C16orf87, CEACAM18, TMCC1, TMCC1, ZFYVE20, NR2C2, MBD4*	*C3orf19*	0.072
19	1	20.8	111.14	*SLC16A7*	SLC16A7	0.46
20	21	45.9	96.53	*UROD, SSR1, MORF4L2, BEST4, PLK3, TCEAL1, SMYD3, KIF2C, HECTD3, EIF2B3, MOSPD2, FANCB, EIF2B3, RAB9B, Glra4, CNST, PLP1, ZSWIM5, BTBD19, AHCY, TCEAL4, PTCH2, TFB2M, Rpl37a-ps1*	*ZSWIM5*	0.097

Supplementary Table 24. Biological Process (BP) GO term enrichment result of genes under selective sweep in wide-ridged finless porpoises. Over-represented GO terms were defined as having at 1.5-fold enrichment and *P* ≤ 0.05 under Fisher's exact test.

ID	Term	Count	Fold Enrichment	Fisher Exact test *P* value
GO:0043462	regulation of ATPase activity	3	32.2	8.80E-05
GO:0019083	viral transcription	6	5.2	1.10E-03
GO:0009607	response to biotic stimulus	3	20.7	3.70E-04
GO:0001666	response to hypoxia	7	3.9	2.10E-03
GO:0006413	translational initiation	6	4.2	3.10E-03
GO:0006614	SRP-dependent cotranslational protein targeting to membrane	5	5.1	2.90E-03
GO:0009440	cyanate catabolic process	2	96.5	1.10E-04
GO:0006364	rRNA processing	7	3.2	7.00E-03
GO:0016266	O-glycan processing	4	6.4	3.50E-03
GO:0090090	negative regulation of canonical Wnt signaling pathway	6	3.6	7.10E-03
GO:0060349	bone morphogenesis	3	10.7	2.70E-03
GO:0000184	nuclear-transcribed mRNA catabolic process, nonsense-mediated decay	5	4.1	8.00E-03
GO:0030855	epithelial cell differentiation	4	5.5	6.00E-03
GO:0030334	regulation of cell migration	4	5.2	7.30E-03
GO:0009060	aerobic respiration	3	8.8	4.70E-03
GO:0070059	intrinsic apoptotic signaling pathway in response to endoplasmic reticulum stress	3	8.8	4.70E-03
GO:0036438	maintenance of lens transparency	2	38.6	1.00E-03
GO:0007631	feeding behavior	3	7.6	7.10E-03
GO:0016311	dephosphorylation	4	4.5	1.20E-02
GO:0019886	antigen processing and presentation of exogenous peptide antigen via MHC class II	4	4.2	1.50E-02
GO:0060071	Wnt signaling pathway, planar cell polarity pathway	4	4.2	1.50E-02

GO:0043525	positive regulation of neuron apoptotic process	3	6.7	1.00E-02
GO:0030336	negative regulation of cell migration	4	4.1	1.70E-02
GO:0006094	gluconeogenesis	3	6.6	1.10E-02
GO:0008542	visual learning	3	6.4	1.10E-02
GO:0042074	cell migration involved in gastrulation	2	24.1	2.90E-03
GO:0006469	negative regulation of protein kinase activity	4	3.9	2.00E-02
GO:0007218	neuropeptide signaling pathway	4	3.8	2.10E-02
GO:0016337	single organismal cell-cell adhesion	4	3.8	2.10E-02
GO:0035176	social behavior	3	6.0	1.30E-02
GO:0000082	G1/S transition of mitotic cell cycle	4	3.8	2.20E-02
GO:0035456	response to interferon-beta	2	21.4	3.70E-03
GO:0016032	viral process	7	2.3	3.70E-02
GO:0043065	positive regulation of apoptotic process	7	2.3	3.70E-02
GO:0006886	intracellular protein transport	6	2.5	3.70E-02
GO:0035455	response to interferon-alpha	2	19.3	4.50E-03
GO:0006476	protein deacetylation	2	19.3	4.50E-03
GO:0032098	regulation of appetite	2	19.3	4.50E-03
GO:0045943	positive regulation of transcription from RNA polymerase I promoter	2	19.3	4.50E-03

Supplementary Table 25. Biological Process (BP) GO term enrichment result of genes under selective sweep in narrow-ridged finless porpoises. Over-represented GO terms were defined as having at 1.5-fold enrichment and P ≤ 0.05 under Fisher's exact test.

ID	Term	Count	Fold Enrichment	Fisher Exact test *P* value
GO:0006413	translational initiation	7	7.5	4.10E-05
GO:0060078	regulation of postsynaptic membrane potential	4	26.8	1.30E-05
GO:0006614	SRP-dependent cotranslational protein targeting to membrane	6	9.4	4.30E-05
GO:0086010	membrane depolarization during action potential	4	21.0	3.60E-05
GO:0019228	neuronal action potential	4	21.0	3.60E-05
GO:0019083	viral transcription	6	7.9	1.10E-04
GO:0000184	nuclear-transcribed mRNA catabolic process, nonsense-mediated decay	6	7.4	1.60E-04
GO:0006814	sodium ion transport	5	9.1	2.20E-04
GO:0006364	rRNA processing	7	4.8	6.40E-04
GO:0034765	regulation of ion transmembrane transport	5	6.6	9.60E-04
GO:0035725	sodium ion transmembrane transport	4	8.1	1.50E-03
GO:0018243	protein O-linked glycosylation via threonine	2	73.6	2.70E-04
GO:0061052	negative regulation of cell growth involved in cardiac muscle cell development	2	73.6	2.70E-04
GO:0006412	translation	6	3.5	7.60E-03
GO:0000082	G1/S transition of mitotic cell cycle	4	5.8	5.10E-03
GO:0035735	intraciliary transport involved in cilium morphogenesis	2	42.1	9.40E-04
GO:2000177	regulation of neural precursor cell proliferation	2	36.8	1.20E-03
GO:0035721	intraciliary retrograde transport	2	29.5	2.00E-03
GO:0048266	behavioral response to pain	2	26.8	2.40E-03
GO:0042147	retrograde transport, endosome to Golgi	3	6.4	1.20E-02
GO:0001967	suckling behavior	2	22.7	3.40E-03
GO:1990126	retrograde transport, endosome to plasma membrane	2	21.0	3.90E-03

Supplementary Table 26. Biological Process (BP) GO term enrichment result of genes under selective sweep in Yangtze River finless porpoises. Over-represented GO terms were defined as having at 1.5-fold enrichment and P ≤ 0.05 under Fisher's exact test.

ID	Term	Count	Fold Enrichment	Fisher Exact test *P* value
GO:0055085	transmembrane transport	6	6.1	4.60E-04
GO:0006836	neurotransmitter transport	3	28.5	1.50E-04
GO:0001822	kidney development	4	11.5	4.10E-04
GO:0006865	amino acid transport	3	21.2	3.80E-04
GO:0015872	dopamine transport	2	70.6	3.30E-04
GO:0007626	locomotory behavior	3	8.8	4.80E-03
GO:0090399	replicative senescence	2	41.2	1.00E-03
GO:0035518	histone H2A monoubiquitination	2	41.2	1.00E-03
GO:0015804	neutral amino acid transport	2	38.0	1.20E-03
GO:1904707	positive regulation of vascular smooth muscle cell proliferation	2	32.9	1.60E-03
GO:0042073	intraciliary transport	2	30.9	1.90E-03
GO:1902895	positive regulation of pri-miRNA transcription from RNA polymerase II promoter	2	24.7	2.90E-03
GO:0070588	calcium ion transmembrane transport	3	6.2	1.20E-02
GO:0003333	amino acid transmembrane transport	2	19.8	4.60E-03
GO:0042787	protein ubiquitination involved in ubiquitin-dependent protein catabolic process	3	4.8	2.40E-02
GO:0006654	phosphatidic acid biosynthetic process	2	14.1	8.80E-03
GO:0051260	protein homooligomerization	3	4.2	3.50E-02
GO:0061025	membrane fusion	2	11.2	1.40E-02
GO:0007269	neurotransmitter secretion	2	9.7	1.80E-02
GO:0043161	proteasome-mediated ubiquitin-dependent protein catabolic process	3	3.6	4.90E-02
GO:0042733	embryonic digit morphogenesis	2	8.8	2.20E-02
GO:0032092	positive regulation of protein binding	2	8.1	2.50E-02
GO:1902600	hydrogen ion transmembrane transport	2	8.1	2.50E-02
GO:0030855	epithelial cell differentiation	2	7.1	3.30E-02
GO:0007265	Ras protein signal transduction	2	7.1	3.30E-02
GO:0032088	negative regulation of NF-kappaB transcription factor activity	2	7.0	3.40E-02
GO:0006813	potassium ion transport	2	6.0	4.40E-02
GO:0016042	lipid catabolic process	2	5.8	4.70E-02

Supplementary Table 27. Biological Process (BP) GO term enrichment result of PSGs in marine narrow ridge finless porpoises. Over-represented GO terms were defined as having at 1.5-fold enrichment and P ≤ 0.05 under Fisher's exact test.

ID	Term	Count	Fold Enrichment	Fisher Exact test *P* value
GO:0042994	cytoplasmic sequestering of transcription factor	3	24.1	2.30E-04
GO:0031290	retinal ganglion cell axon guidance	3	16.5	7.50E-04
GO:0035335	peptidyl-tyrosine dephosphorylation	5	5.3	2.60E-03
GO:0006906	vesicle fusion	4	7.1	2.50E-03
GO:0031440	regulation of mRNA 3'-end processing	2	69.5	2.70E-04
GO:2001170	negative regulation of ATP biosynthetic process	2	69.5	2.70E-04
GO:0021914	negative regulation of smoothened signaling pathway involved in ventral spinal cord patterning	2	69.5	2.70E-04
GO:0061364	apoptotic process involved in luteolysis	2	69.5	2.70E-04
GO:0030308	negative regulation of cell growth	5	4.3	6.20E-03
GO:0006378	mRNA polyadenylation	3	11.2	2.40E-03
GO:0048662	negative regulation of smooth muscle cell proliferation	3	10.8	2.60E-03
GO:0006611	protein export from nucleus	3	10.4	2.90E-03
GO:0006816	calcium ion transport	4	5.5	6.10E-03
GO:0097500	receptor localization to nonmotile primary cilium	2	41.7	9.00E-04
GO:0051898	negative regulation of protein kinase B signaling	3	8.5	5.30E-03
GO:0001657	ureteric bud development	3	8.2	5.70E-03
GO:0048681	negative regulation of axon regeneration	2	34.8	1.30E-03
GO:1901621	negative regulation of smoothened signaling pathway involved in dorsal/ventral neural tube patterning	2	34.8	1.30E-03
GO:0030521	androgen receptor signaling pathway	3	7.6	7.10E-03
GO:0000398	mRNA splicing, via spliceosome	6	2.8	2.00E-02
GO:1903546	protein localization to photoreceptor outer segment	2	29.8	1.90E-03
GO:2000574	regulation of microtubule motor activity	2	29.8	1.90E-03
GO:0001561	fatty acid alpha-oxidation	2	29.8	1.90E-03
GO:0032870	cellular response to hormone stimulus	3	7.0	9.10E-03
GO:0031175	neuron projection development	4	4.2	1.60E-02
GO:1903076	regulation of protein localization to plasma membrane	2	26.1	2.50E-03
GO:0006464	cellular protein modification process	4	4.0	1.80E-02
GO:0010923	negative regulation of phosphatase activity	3	6.1	1.30E-02
GO:0035556	intracellular signal transduction	8	2.1	4.10E-02
GO:0007507	heart development	5	2.8	3.20E-02
GO:1903779	regulation of cardiac conduction	3	5.6	1.70E-02
GO:0070588	calcium ion transmembrane transport	4	3.5	2.80E-02
GO:0006661	phosphatidylinositol biosynthetic process	3	5.4	1.80E-02

GO:0035385	Roundabout signaling pathway	2	17.4	5.70E-03
GO:0009190	cyclic nucleotide biosynthetic process	2	17.4	5.70E-03
GO:0021670	lateral ventricle development	2	17.4	5.70E-03
GO:0001933	negative regulation of protein phosphorylation	3	5.1	2.10E-02
GO:0042384	cilium assembly	4	3.4	3.20E-02
GO:0016540	protein autoprocessing	2	16.0	6.60E-03
GO:0006369	termination of RNA polymerase II transcription	3	4.9	2.40E-02
GO:0014912	negative regulation of smooth muscle cell migration	2	14.9	7.70E-03
GO:0047496	vesicle transport along microtubule	2	14.9	7.70E-03
GO:0043087	regulation of GTPase activity	3	4.8	2.50E-02
GO:0007405	neuroblast proliferation	2	13.0	1.00E-02
GO:0030518	intracellular steroid hormone receptor signaling pathway	2	13.0	1.00E-02
GO:0006182	cGMP biosynthetic process	2	13.0	1.00E-02
GO:0001501	skeletal system development	4	3.0	4.30E-02
GO:0048738	cardiac muscle tissue development	2	12.3	1.10E-02
GO:0021542	dentate gyrus development	2	12.3	1.10E-02
GO:0000724	double-strand break repair via homologous recombination	3	4.2	3.40E-02
GO:1903861	positive regulation of dendrite extension	2	11.6	1.30E-02
GO:0048854	brain morphogenesis	2	11.6	1.30E-02
GO:0010596	negative regulation of endothelial cell migration	2	11.6	1.30E-02
GO:0045879	negative regulation of smoothened signaling pathway	2	11.0	1.40E-02
GO:0001843	neural tube closure	3	4.1	3.80E-02
GO:0001892	embryonic placenta development	2	10.4	1.50E-02
GO:2000463	positive regulation of excitatory postsynaptic potential	2	10.4	1.50E-02
GO:0019226	transmission of nerve impulse	2	10.4	1.50E-02
GO:0007018	microtubule-based movement	3	3.9	4.30E-02
GO:0006699	bile acid biosynthetic process	2	9.9	1.70E-02
GO:0050772	positive regulation of axonogenesis	2	9.9	1.70E-02
GO:0000132	establishment of mitotic spindle orientation	2	9.9	1.70E-02
GO:0043687	post-translational protein modification	2	9.5	1.90E-02
GO:0048754	branching morphogenesis of an epithelial tube	2	9.1	2.00E-02
GO:2001235	positive regulation of apoptotic signaling pathway	2	8.3	2.40E-02
GO:0097352	autophagosome maturation	2	8.3	2.40E-02
GO:0060976	coronary vasculature development	2	8.3	2.40E-02
GO:0030534	adult behavior	2	8.0	2.60E-02
GO:0006891	intra-Golgi vesicle-mediated transport	2	7.7	2.70E-02
GO:0001656	metanephros development	2	7.7	2.70E-02
GO:0035987	endodermal cell differentiation	2	7.7	2.70E-02
GO:0032008	positive regulation of TOR signaling	2	7.7	2.70E-02
GO:0006910	phagocytosis, recognition	2	7.4	2.90E-02
GO:0032007	negative regulation of TOR signaling	2	7.4	2.90E-02
GO:0006810	transport	6	1.8	1.20E-01

GO:0051301	cell division	6	1.8	1.20E-01
GO:0021510	spinal cord development	2	7.0	3.30E-02
GO:0030513	positive regulation of BMP signaling pathway	2	6.7	3.50E-02
GO:0016567	protein ubiquitination	6	1.7	1.30E-01
GO:0051480	regulation of cytosolic calcium ion concentration	2	6.5	3.80E-02
GO:0009306	protein secretion	2	6.5	3.80E-02
GO:0006468	protein phosphorylation	7	1.6	1.50E-01
GO:0007420	brain development	4	2.2	1.10E-01
GO:0045494	photoreceptor cell maintenance	2	6.1	4.20E-02
GO:0043588	skin development	2	6.1	4.20E-02
GO:0017158	regulation of calcium ion-dependent exocytosis	2	6.0	4.40E-02
GO:0007611	learning or memory	2	5.8	4.70E-02
GO:0007399	nervous system development	5	1.8	1.40E-01

Supplementary note 1

Samples information

There are two forms, i.e., 'narrow-ridge' and 'wide-ridge' formed finless porpoises, which were classified according to tubercles distribution on their back ridge. The individual with width of the tuberculed area larger than 4 cm and more than 10 rows of tubercules were assigned as 'wide-ridged' form, whereas the rest samples with the width of tuberculed area less than 0.7 cm and there were only 3 and 5 rows of tubercules were recognized as 'narrow-ridged' form[1-2]. Previous studies and our previous fieldwork have shown could distinguish two forms of finless porpoises reliably[3]. For *de novo* sequencing and assembly, an adult male finless porpoise (narrow-ridge form) was collected from the Yangtze River in Xiaguan, Nanjing of Jiangsu Province, which was stranded and already dead for some unknown reason. A total of additional 48 finless porpoise individuals were collected for whole genomic resequencing. The sample examined in this study contained 13 individuals from the Yangtze River (Anqing, Tongling, Nanjing), 15 individuals from the Yellow sea and East China Sea (Lvsi, Ningbo), and 20 individuals from the South China Sea (Pingtan, Dongshan). Voucher specimens were preserved at Jiangsu Key Laboratory for Biodiversity and Biotechnology, College of Life Sciences, Nanjing Normal University. Total genomic DNA from muscles or skeleton samples was extracted by using stand Phenol-chloroform method.

Genome sequencing

The whole genome shotgun strategy and the next-generation sequencing technologies on the Illumina HiSeq 2000 platform were used to sequence the genome of a finless porpoise. Multiple insert sizes (250bp, 500bp, 800bp, 2Kb, 5Kb, 10Kb, 20Kb, 40Kb) were designed to build sequencing libraries and a total of 484.88 Gb reads were generated for the de novo assembly. To reduce impact of sequencing errors, there are several correction and filter criteria for the raw data from Illumina-Pipeline: (1) 5% reads bases with N were filtered. (2) Reads with more than 40% of low quality bases in small insert size libraries and 60% in larger than 800bp insert size libraries were filtered. (3) Reads with adapter contamination which aligned to the adapter sequence (match length ≥10bp, mismatch ≤ 3) were filtered. (4) Filter

reads with small insert size when read1 and read2 overlapped ≥10 bp, mismatch ≤10%. (5) Filtered reads with PCR duplicated (read1 and read2 were totally same). Using these high-quality reads, genome size of finless porpoise is estimated to be 2.488 Gb using a 17-mer analysis[4]. Particularly, sequencing errors could easily bring up low frequency k-mer. To avoid this, error correction procedure was used to deleted 0.59% reads and 2.16% bases which the frequency of 17 k-mer lower than 10.

Genome assembly

After filtering and correction, SOAP*denovo*-2.04 (http://soap.genomics.org.cn)[5] was employed to assembly the finless porpoise genome using qualified reads. For constructing the contig, short reads from fragmented small insert-size libraries were assembled into contig base on overlap information. The total contig size and N50 of finless porpoise were 2.28Gb and 26.7 Kb, respectively. Usable reads were realigned to the contig sequences, then paired-end relationship between pairs of contigs was used to construct scaffolds by linking contigs. We calculated and weighted with the rate of consistent and conflicting paired ends before constructing the scaffolds in a stepwise manner from the short–insert size paired ends to the long–insert size paired ends. This time, the total scaffold size and N50 were 2.30Gb and 6.33Mb, respectively. To fill the intra-scaffold gaps, we used the paired-end information to retrieve read pairs with one end uniquely aligned to a contig and the other end in the gap region. At last, a local assembly was achieved for these collected reads.

At present, the comparison of assembly statistics between finless porpoises and other six cetaceans that with whole genome sequenced (i.e. *Balaena mysticetus*, http://www.bowhead-whale.org; *Balaenoptera acutorostrata*, BalAcu1.0, NCBI; *Physeter catodon*, Physeter_macrocephalus-2.0.2, NCBI; *Lipotes vexillifer_v1*, NCBI; *Orcinus orca*, Oorc_1.1, NCBI; *Tursiops truncatus*, Ttru_1.4, NCBI) were shown in Supplementary Table 4.

Assessment of genome assembly

The scatter graph of the distribution of GC content against sequencing depth shown that the distribution of GC content for finless porpoise was largely above 60× and relatively concentrated (Supplementary Figure 1). For region with lower average

depth (30-60×) of the scatter plot, it is likely to be Y chromosome, which has half the sequencing depth of autosomes. And there was no obvious difference with an average GC content of 41.1% among the seven cetaceans that had been fully sequenced.

Then, the transcriptome data was used to measure quality of the finless porpoise genome assembly. The total RNA from blood cells of two finless porpoise were extracted with TRIZOL (Invitrogen) and then reversed transcribed into cDNA using the PrimeScript™ RT reagent Kit (Takara), respectively. After sequencing using HiSeq2000 and assembling using Trinity[6], we generated 72, 056 transcripts to align back to the genome by using BLAT[7] with default parameters except an identity cutoff of 90%. More than 98% of assembly could be mapped successfully by the unigenes (Supplementary Table 6). Additionally, RNA-seq reads were aligned to the finless porpoise reference genome using TopHat v2.0.7[8] with default parameters. The best quality blood sample achieved 81.71% mapping rate (Supplementary Table 5).

Finally, we further used protein coding genes of the common bottlenose dolphin and baiji and mapped them to the finless porpoise genome assembly by BLAT[7] to make sure the quality of our assembly (Supplementary Table 7).

Detections of heterozygous SNPs

The heterozygosity rate of finless porpoises was estimated (Supplementary Figure 1). First, the high-quality reads were realigned to the assembly genomes by the help of BWA[9]. Then SNP calling was done by SOAPsnp 1.03[5] to achieve ~ 2.3 M heterozygous SNPs for the finless porpoise genome with a high-confidence (i.e. the coverage depth ≥ 10 and ≤ 250, the genotype quality ≥ 20, copy number ≤ 2 and the distance of adjacent SNPs ≥ 5), representing a heterozygous SNP rate 0.09% in the finless porpoise genome.

Annotation of protein-coding genes

To build our protein coding genes dataset, homolog prediction and *de novo* prediction were carried out. It was Integrated two annotation processes by program GLEAN (http://sourceforge.net/projects/glean-gene/) into a non-redundancy and more complete protein coding gene dataset. For homolog prediction, the homolog sequence of species (human, dolphin, baiji, minke whale, dog, pig and cow) were

downloaded from Ensembl (version65) and then mapped to the genome by tBLASTn with E-value cutoff of 1×10^{-5}. After that, GENEWISE[10] was used to generate gene structure through aligned sequence and its query protein. Augustus[11] and Genscan[12] were used in *de novo* prediction (Supplementary Fig. 2). After GLEAN procedures we successfully constructed a non-redundant gene set, and the final protein coding gene set were totally 22,014 (Supplementary Table 8).

Additionally, function annotation of predicted genes were assigned according to the BLASTP with E-value cutoff of 1×10^{-5}, this best match of the alignment to the SwissProt and Translated EMBL Nucleotide Sequence Data Library (TEMBL) databases[13]. Motifs and domains were determined by searches in InterProScan[14] of the sequences against publicly available databases, including Pfam, PRINTS, PROSITE, ProDom and SMART. The Gene Ontology[15] IDs for each gene were achieved from the corresponding InterPro entry. We also mapped finless porpoise reference genes to KEGG pathway databases and identified the best match for each gene (Supplementary Table 9).

Annotation of non-coding RNAs

Four types of non-coding RNAs (ncRNAs), including transfer RNAs (tRNAs), ribosomal RNAs (rRNAs), micro RNAs (miRNAs) and small nuclear RNAs (snRNAs), were also predicted and annotated within finless porpoise assembly (Supplementary Table 10). According to the sequence structure of tRNA, tRNAscan-SE[16] with eukaryote parameters was employed to predict tRNA in finless porpoise genome. For the conservative rRNA, our genome was aligned with reference human full-length rRNAs by BLASTN with a parameter of E value ≤1e-5, identity ≥5% and the matched length ≥50bp. The snRNAs and miRNAs were annotated using a two-steps method: after aligning with BLAST, INFERNAL[17] was used to search for putative sequences in the Rfam database (release 9.1)[18].

Annotation of transposable elements (TE)

Annotation of transposable elements (TEs) in the finless porpoise assembly were integrated two principle methods: 1) based on homology which starts to identify known TEs by RepeatMasker program, against the Repbase database (version 16.10) (http://www.repeatmasker.org) of known repeats, then aligned the genome

sequence to the TE protein database used RepeatProteinMask by WU-BLASTX to identify TEs; 2) *de novo* method that used program RepeatModeler based on sequence alignment (http://www.repeatmasker.org). The tandem repeats were found in the genomic sequence data using the software Tandem Repeats Finder (version 4.04)[19] with the parameter 'Match=2, Mismatch=7, Delta=7, PM=80, PI=10, Minscore=50, and MaxPeriod=2000'. Summarizing all methods, 45.18% of finless porpoise genome was repeats (Supplementary Table 11). The major classification of TEs was also calculated (Supplementary Fig. 3 and Supplementary Table 12).

Identification of synteny

Using LASTZ[20], the syntenic region among finless porpoise and other two cetaceans (minke whale and baiji) and cow was assessed with parameters 'T=2, C=2, H=2200, Y=3400, L=6000 and K=2200' (Supplementary Table 13).

Supplementary note 2

Gene family cluster and orthology relationship

The Treefam methodology[21] were used to define a gene family (a group of genes that descended from a single gene in the last common ancestor of the considered species) relationships among finless porpoises, six cetaceans (*Lipotes vexillifer, Tursiops truncatus, Orcinus orca, Balaenoptera acutorostrata, Balaena mysticetus, Physeter catodon*), 10 terrestrial mammals (*Bos taurus, Canis lupus familiaris, Ovis aries, Sus scrofa, Equus caballus, Homo sapiens, Felis catus, Pteropus vampyrus, Erinaceus europaeus, Sorex araneus*). For genes with alternative splicing variants, the longest transcripts were selected to represent the genes. And an all-against-all BLASTP was applied to determine the similarities between genes with the e-value of 1e-7 and conjoined fragmental alignments for each gene pair by Solar (Supplementary Fig. 2). We assigned a connection (edge) between any two nodes (genes) if more than 1/3 of the region aligned to both genes. An H-score that ranged from 0 to 100 was used to weigh the similarity (edge). In particular, for two genes, G1 and G2, the H-score was defined as a score (G1G2)/max (score(G1G1), score(G2G2)) (the score here is the raw Blast score). Then, the extraction of gene families (clustering by Hcluster_sg) was used the average distance for the hierarchical clustering algorithm, with requiring the

minimum edge weight (H-score) to be larger than 5, and the minimum edge density (total number of edges/theoretical number of edges) to be larger than 1/3. A Venn diagrams has been used to show the distribution of shared and unique gene families in seven sequenced cetaceans (Supplementary Fig. 2).

Expansion/contraction of gene families

CAFÉ[22] was used to calculate the gene family gain and lose over a phylogenetic tree with divergence time based on the model of random birth and death. One important parameter λ (lambda) which describes both the gene birth (λ) and death (μ= -λ) rate across all branches in tree for all gene families was estimated using maximum likelihood method as implemented in RAxML software[23]. For each gene family, the accelerated rate of gain/loss was set to be with conditional *P* value less than threshold 0.05.

Positively selected genes

Using the 3,911 single-copy genes shared by the finless porpoise, 6 other cetaceans and 10 terrestrial mammals, positively selected genes (PSGs) were identified in the finless porpoise. The branch-site model[24] was used to detect positive selection along a target branch. We compared Model A1 (neutrally or under purifying selection) with Model A (positive selection) and p-values were computed using the χ2 statistic. PRANK (http://wasabiapp.org/software/prank/) and Gblocks[25] was used to make alignment and remove potentially unreliable regions. A total of 57 PSGs were identified in the finless porpoise, and five (*AHI1*, *CC2D2A*, *FANCD2*, *STT3A* and *TTBK2*) are involved in the development of cerebellum. Notably, mutations in *AHI1* and *CC2D2A* are associated with Joubert syndromes 3 and 9 characterized by a disorder of balance and coordination due to the malformed brain stem and cerebellar vermis[26,27]. Homozygous *STT3A* mutations cause many congenital disorders, including microcephaly and cerebellar atrophy[28]. Rapidly evolving cerebellum development genes in porpoise may possibly be connected to the strikingly smaller volumes of the cerebellum (less than 80 mL) and vermis (5.80 mL) in harbor porpoise compared to delphinids (typically, for bottlenose dolphin, cerebellum is ~291 mL and vermis is 22.42 mL)[29].

Supplementary note 3

Population resequencing and SNP calling

After sequencing process on Illumina HiSeq 2000 platform, 1865.1Gb high quality pair-end reads (90bp) from 48 finless porpoise individuals were mapped to the *denovo* genome with Burrows-Wheeler Aligner (BWA)[9]. After the alignment, SAMtools[30], Picard pack tools (https://broadinstitute. github.io/picard/) and Genome Analysis Toolkit (GATK, version 2.4-9)[31] were employed to call SNPs and filter at population scale. As there is a low-quality alignment around the indel region, two steps of realignment were implemented in GATK: 'RealignerTargetCreator' package was used to identify regions which needs realignment in the first step. The second step with 'IndelRealigner' performed realignment the regions found in the first step. SNPs were also annotated by SNPEFF[32] and summarized characteristic of SNPs by a customized Perl script. This annotation for the whole SNPs set was used for subsequent population genomic analyses.

Supplementary note 4

Phylogenetic tree and Population structure

Phylogeny tree of finless porpoises were reconstructed based on neighbor-join method by TreeBeST[33]. The program FRAPPE[34] was utilized to infer population structure and ancestry information. We did not assume any prior information about their ancestry. Additional, used ADMIXTURE[35] run 10,000 iterations and pre-defined the number of cluster, *K*, from 2 to 5. The cross-validation test[36] were used to find the best *K* value. We performed a PCA following the procedure as reported. The eigenvector decomposition of the transformed genotype data was performed using the R function Eigen, and the significance of the eigenvectors was determined with a Tracey-Widom test, implemented in the program twstats provided by the EIGENSOFT software3.2[37].

LD analysis

LD was calculated based on the SNPs with minor allele frequency (MAF) greater than 0.05 using Haploview software[38]. Three populations were separated, and SNPs in each population were extracted to perform the analysis. These parameters were '−n −pedfile −info −log −minMAF 0.05 −hwcutoff 0.001 −dprime −memory 2096'. After that, values for the *r*2 and *D*' statistics were obtained.

Demographic history reconstruction

The Pairwise Sequential Markovian Coalescent (PSMC) model could be used to inference ancestral effective population size (*N*e) based on information from inter-chromosomal genetic differences within a single individual[39]. We applied this model to infer the Yangtze finless porpoise, ocean narrow-ridge form and ocean wide-ridge form in our data set to investigate their respective demographic histories. The psmcfa format input files was generated follow the authors instruction, using 100 bp bins and accounting for uncallable sites as required by the software usage specification. PSMC was run with the command 'PSMC -N25 -t15 -r5 -p 4+25*2+4+6'. Results were scaled using an assumed mutation 1.14e-8 per bp per generation and a generation time of 8 years.

The Multiple sequential Markovian coalescent (MSMC) model[40] is an HMM along multiple phased haplotypes which were used to infer effective population size and population separation over time from now to 50000 years ago. Here only autosomes were used and the haplotype were phased based on all the sequenced samples with SHAPEIT[41]. Scaffolds in finless porpoise assembly that shown sytenic relationships to cow X chromosome (determined by Lastz) has not been included in this analysis and previous PSMC model.

Supplementary note 5

Population selection analysis

Composite likelihood ratio (CLR)[42] estimated for each SNP using SweepFinder2[43] for wide and narrow ridged finless porpoises, respectively. The top 20 genome 'peaks' with the CLR higher than 0.2% of CLR were picked out as candidate selective sweep regions, and genes in these regions (within 1Mb flanking the sweep region) are identified as putative genes under selection.

Within narrow-ridged finless porpoise, we further performed the XP-EHH[44] test in both directions and for all SNPs. Outlying XP-EHH scores (top 0.1%) are potentially indicative of selection in a particular population. In order to reduce our false positive rate by choosing to declare a region significant only when a cluster of nearby SNPs has outlying XPEHH scores, we divide the genome into 50kb windows with a step size of 5 kb, and identify candidate regions for selection as those in which more than 0.1 fraction of SNPs within them have an XPEHH score above cutoff value of top 1%.

References:

1. Gao, A.L. & Zhou, K.Y. Geographical variation of external measurements and three subspecies of *Neophocaena phocaenoides*in Chinese waters. Acta. Theriol. Sin.**15**, 81–92 (1995).
2. Jefferson, T.A. & Wang, J.Y. Revision of the taxonomy of finless porpoises (genus *Neophocaena*): The existence of two species. J. Mar. Anim. Ecol. **4**, 3-16 (2011).
3. Pilleri, G. & Gihr, M. On the taxonomy and ecology of the finless black porpoise, *Neophocaena* (Cetacea, Delphinidae). Mammalia **39**, 657–673 (1975).
4. Li, R., et al. The sequence and de novo assembly of the giant panda genome. Nature **463**, 311-317 (2010)
5. Li, R., Li, Y., Kristiansen, K., & Wang, J. SOAP: short oligonucleotide alignment program. Bioinformatics **24**, 713-714 (2008).
6. Grabherr, M. G., et al. Full-length transcriptome assembly from RNA-Seq data without a reference genome. Nat. Biotechnol. **29**, 644-652 (2011).
7. Kent, W. J. BLAT--the BLAST-like alignment tool. Genome Res. **12**, 656-664. (2002).
8. Trapnell, C., Pachter, L. & Salzberg, S. L. TopHat: discovering splice junctions with RNA-Seq. Bioinformatics **25**, 1105-1111 (2009).
9. Li, H. & Durbin, R. Fast and accurate short read alignment with Burrows–Wheeler transform. Bioinformatics **25**, 1754-1760 (2009).
10. Birney, E., Clamp, M. & Durbin, R. GeneWise and genomewise. Genome Res. **14**, 988-995 (2004).
11. Stanke, M., et al. AUGUSTUS: ab initio prediction of alternative transcripts. Nucleic Acids Res. **34**, W435-W439 (2006).
12. Burge, C. & Karlin, S. Prediction of complete gene structures in human genomic DNA. J Mol Biol. **268**, 78-94 (1997).
13. Bairoch, A. & Apweiler, R. The SWISS-PROT protein sequence database and its supplement TrEMBL in 2000. Nucleic Acids Res. **28**, 45-48 (2000).

14. Zdobnov, E.M. & Apweiler, R. InterProScan–an integration platform for the signature-recognition methods in InterPro. Bioinformatics **17**, 847-848 (2001).

15. Ashburner, M. et al. Gene Ontology: tool for the unification of biology. Nat. Genet. **25**, 25-29 (2000).

16. Lowe, T. M. & Eddy, S. R. tRNAscan-SE: a program for improved detection of transfer RNA genes in genomic sequence. Nucleic Acids Res. **25**, 955-964 (1997).

17. Nawrocki, E. P., Kolbe, D. L., & Eddy, S. R. Infernal 1.0: inference of RNA alignments. Bioinformatics **25**, 1335-1337 (2009).

18. Griffiths-Jones, S., et al. Rfam: annotating non-coding RNAs in complete genomes. Nucleic Acids Res. **33**, D121-D124 (2005).

19. Benson, G. Tandem repeats finder: a program to analyze DNA sequences. Nucleic Acids Res. **27**, 573-580 (1999).

20. Harris, R.S. Improved pairwise alignment of genomic DNA. Ph.D. Thesis, The Pennsylvania State University. (2007).

21. Li, H., et al. TreeFam: a curated database of phylogenetic trees of animal gene families. Nucleic Acids Res. **34(suppl 1)**, D572-D580 (2006).

22. De Bie, T., Cristianini, N., Demuth, J. P. & Hahn, M. W. CAFE: a computational tool for the study of gene family evolution. Bioinformatics **22**, 1269-1271. (2006).

23. Stamatakis, A. RAxML version 8: a tool for phylogenetic analysis and post-analysis of large phylogenies. Bioinformatics **30**, 1312-1313 (2014).

24. Zhang, J., Nielsen, R. & Yang, Z. Evaluation of an improved branch-site likelihood method for detecting positive selection at the molecular level. Mol. Biol. Evol. **22**,

2472-2479. (2005).

25. Castresana, J. Selection of conserved blocks from multiple alignments for their use in phylogenetic analysis. Mol. Biol. Evol. **17**, 540-552 (2000).

26. Ferland, R.J., et al. Abnormal cerebellar development and axonal decussation due to mutations in AHI1 in Joubert syndrome. Nat. Genet. **36**, 1008-1013 (2004)

27. Noor, A., et al. CC2D2A, encoding a coiled-coil and C2 domain protein, causes autosomal-recessive mental retardation with retinitis pigmentosa. Am. J. Hum. Genet. **82**, 1011-1018 (2008).

28. Shrimal, S., Ng, B.G., Losfeld, M.E., Gilmore, R. & Freeze, H.H. Mutations in STT3A and STT3B cause two congenital disorders of glycosylation. Hum. Molec. Genet. **22**, 4638-4645 (2013).

29. Maseko, B.C., Spocter, M.A., Haagensen, M. & Manger, P.R. Elephants have relatively the largest cerebellum size of mammals. Anat. Rec. (Hoboken) **295**, 661-672 (2012).

30. Li, H., et al. The sequence alignment/map format and SAMtools. Bioinformatics **25**, 2078-2079 (2009).

31. McKenna, A. et al. The Genome Analysis Toolkit: a MapReduce framework for analyzing next-generation DNA sequencing data. Genome Res. **20**, 1297-1303 (2010).

32. Cingolani, P., et al. A program for annotating and predicting the effects of single nucleotide polymorphisms, SnpEff: SNPs in the genome of Drosophila melanogaster strain w1118; iso-2; iso-3. Fly, **6**, 80-92 (2012).

33. Vilella, A. J., et al. EnsemblCompara GeneTrees: Complete, duplication-aware phylogenetic trees in vertebrates. Genome Res. **19**, 327-335 (2009).

34. Tang, H., Peng, J., Wang, P. & Risch, N. J. Estimation of individual admixture: analytical and study design considerations. Genet. Epidemiol. **28**, 289-301 (2005).

35. Alexander, D. H., Novembre, J. & Lange, K. Fast model-based estimation of ancestry in unrelated individuals. Genome Res. **19**, 1655-1664 (2009).

36. Alexander, D. H. & Lange, K. Enhancements to the ADMIXTURE algorithm for individual ancestry estimation. BMC Bioinformatics **12**, 246 (2011).

37. Patterson, N., Price, A. L. & Reich, D. Population structure and eigen analysis. PLoS genet. **2**, e190 (2006).

38. Barrett, J. C., Fry, B., Maller, J. D. M. J. & Daly, M. J. Haploview: analysis and visualization of LD and haplotype maps. Bioinformatics **21**, 263-265 (2005).

39. Li, H., & Durbin, R. Inference of human population history from individual whole-genome sequences. Nature **475**, 493-496 (2011).

40. Schiffels, S. & Durbin, R. Inferring human population size and separation history from multiple genome sequences. Nat. Genet. **46**, 919-925. (2014).

41. Delaneau, O., Marchini, J. & Zagury, J. F. A linear complexity phasing method for thousands of genomes. Nature Methods **9**, 179-181 (2012).

42. Nielsen, R. et al. Genomic scans for selective sweeps using SNP data. Genome Res. **15**, 1566-1575 (2005).

43. DeGiorgio, M., Huber, C.D., Hubisz, M.J., Hellmann, I. & Nielsen, R. SweepFinder2: increased sensitivity, robustness and flexibility. Bioinformatics **32**, 1895-1897 (2016).

44. Sabeti, P. C., et al. Genome-wide detection and characterization of positive selection in human populations. Nature **449**, 913-918 (2007).
