# Supplementary material for: Population genomics of finless porpoises reveal an incipient cetacean species adapted to freshwater
Source: Nat Commun. 2018 Apr 10;9:1276. doi: 10.1038/s41467-018-03722-x (PMC5893588; doi:10.1038/s41467-018-03722-x)
Supplement: Supplementary file 2 — Description of Additional Supplementary Files(PDF 161 kb) [file 41467_2018_3722_MOESM2_ESM.pdf]

Legend for Supplementary Data:

Supplementary Data 1: List of genes under selective sweep identified in Yangtze River finless porpoises using XP-EHH method.

Supplementary Data 2: List of genes under selective sweep identified in marine narrow ridge finless porpoises (Yellow population) using XP-EHH method.
